# Automated cardiac strain estimation from short-axis DENSE and Cine MR images

**DOI:** 10.1007/s10237-026-02120-3

**Published:** 2026-09-15

**Authors:** Mohammad Naqizadeh Jahromi, Rodrigo Menna Costa, Augusto Delavald Marques, Luigi Wellner, Ariel J. Hannum, Zhan-Qiu Liu, Dazhong Wu, Daniel B. Ennis, Luigi E. Perotti

**Affiliations:** 1 Department of Mechanical and Aerospace Engineering, University of Central Florida, 12760 Pegasus Drive, Orlando, 32816 FL USA; 2 Department of Radiology, Stanford University, 300 Pasteur Drive, Stanford, 94305 CA USA; 3 Division of Radiology, Veterans Administration Health Care System, 3801 Miranda Avenue, Palo Alto, 94304 CA USA; 4 Cardiovascular Institute, Stanford University, 265 Campus Drive, Stanford, 94305 CA USA; 5 Department of Bioengineering, Stanford University, 443 Via Ortega, Stanford, 94305 CA USA; 6 Biionix Cluster, University of Central Florida, 4000 Central Florida Blvd, Orlando, 32816 FL USA

**Keywords:** Cardiac strains, Cardiac kinematics, DENSE MRI, Cine MRI, nnU-Net

## Abstract

Cardiac strains provide significant information to evaluate cardiac performance. They can be evaluated using full field voxel-wise displacements measured, for example, using displacement encoding with stimulated echoes (DENSE) magnetic resonance imaging (MRI) or from features and textures that are measured using Cine MRI. While DENSE MRI provides a more complete description of myocardial motion, it remains challenging to acquire and its adoption is not widespread. In contrast, Cine MRI is routinely acquired, and Cine-based cardiac strains could be more easily adopted in a clinical setting. To evaluate the feasibility of automatically estimating cardiac strains from Cine MRI, we compare slice-wise circumferential, longitudinal, and radial strains obtained with three mid-ventricular models: a two-slice DENSE model, a one-slice DENSE model, and a one-slice Cine model. Strains are computed after automatic image segmentation, and the models are evaluated using a computational deforming phantom and data acquired in forty (N=40) healthy volunteers. When evaluated against the computational phantom, all models perform well in terms of circumferential strains, while differences are present in longitudinal and radial strains depending on model and location. When applied to volunteer data, the one-slice Cine model agrees reasonably well with the DENSE-based models in terms of endocardial circumferential strains while, overall, it leads to higher (in magnitude) radial and longitudinal strains, and lower (in magnitude) epicardial circumferential strains. The DENSE models lead to nearly identical estimates for circumferential and radial strains, while longitudinal strains are not correctly estimated by the one-slice DENSE model paired with automatic segmentation. The strain differences are discussed both group-wise and at the individual subject level.

## Introduction

Cardiac performance is routinely estimated by measuring the ejection fraction (EF) in the clinic, but EF changes relatively late in the course of disease and only reflects global functional change. On the other hand, global and regional cardiac strains improve the assessment of cardiac function and provide additional information regarding risk stratification, outcome prediction, and early assessment (Halliday et al. 2021; Zhang et al. 2014; Brady et al. 2023; Potter and Marwick 2018). In this context, global radial, circumferential, and especially longitudinal cardiac strains have been evaluated as diagnostic tools and outcome predictors (e.g., Marwick et al. 2019; Zhang et al. 2019; Park et al. 2018; Olsen et al. 2021): Improving the ability to automatically compute them from routinely acquired images may enhance their integration in clinical practice.

Cardiac strains can be estimated from images acquired using different medical imaging modalities, including echocardiography and magnetic resonance imaging (MRI) (Voigt and Cvijic 2019; Smiseth et al. 2025), and computed tomography (Buss et al. 2014; McVeigh et al. 2018). In this work, we focus on estimating cardiac strains computed from MRI, which allows one to compare strains computed from 3D voxel-wise displacements to strains estimated from surface motion only. The 3D voxel-wise displacements are measured via displacement encoding with stimulated echoes (DENSE) MRI (Aletras et al. 1999), while surface motion is extracted from balanced steady-state free precession (bSSFP) Cine MRI, which is a fast and widely used MRI sequence (Oppelt et al. 1986; Zur et al. 1988; Kramer et al. 2020). In particular, we will consider three models: (1) a two-slice DENSE model estimating strains from a complete set of 3D voxel-wise displacements computed from two DENSE MRI short-axis slices; (2) a one-slice DENSE model (lacking information about displacements gradients along the longitudinal axis with respect to the two-slice DENSE model); and (3) and a one-slice Cine model estimating slice-wise strains from surface motion derived from one Cine MRI short-axis slice.

Several other approaches have computed cardiac strains from cine MRI after estimating motion based on image registration from a chosen reference to a deformed configuration (e.g., López et al. 2023; Álvarez-Barrientos et al. 2026; Genet et al. 2018; Bistoquet et al. 2008; Morales et al. 2021; Mukherjee et al. 2025). These works leverage different image registration strategies based on physics-informed neural networks (e.g., accounting for low myocardial compressibility and contraction along the myofiber directions), image similarity indices, methods to penalize deviation from mechanical equilibrium, deep learning models, and super resolution reconstruction. In this work, we propose a simpler approach that can be applied to an individual short-axis (SA) Cine slice to estimate slice-wise circumferential, longitudinal, and radial strains.

DENSE MRI, used for comparison in the current work, directly encodes tissue motion into the phase of the complex-valued MR data (Spottiswoode et al. 2007). After spatial and temporal phase unwrapping and subsequent processing (e.g., using trajectory tracking Spottiswoode et al. 2007), the voxel-wise phase information is transformed into voxel-wise Lagrangian displacement information, from which cardiac strains can be computed after displacement interpolation and differentiation. In this context, the DENSE-Analysis toolbox remains a popular, open-source, and publicly available software for displacement and strain computation from DENSE imaging data (Spottiswoode et al. 2007; Gilliam and Suever 2021). Although the DENSE-Analysis toolbox remains an important tool to process DENSE data, other approaches based on deep learning (DL) for image segmentation and phase unwrapping (Ghadimi et al. 2021), and advanced frameworks for displacement calculations (Ghadimi et al. 2023) have been proposed in recent years. In this work, given its wide use and public availability, we use the DENSE-Analysis toolbox to compute Lagrangian displacements, which are then further processed to compute cardiac strains.

This work extends our previous study (Marques et al. 2025) presented at the Functional Imaging and Modeling of the Heart (FIMH) international conference in three main aspects: (1) Instead of using a manual segmentation of the DENSE imaging data, we use an nnU-Net model to segment the myocardium. A separately trained nnU-Net model is also employed to segment the Cine images. This modification improves robustness since user-dependent decisions are not necessary in the DENSE data segmentation, which is often challenging and time-consuming. (2) We expand the subject cohort from ten (N=10) to forty (N=40) subjects, improving the generalizability of our findings. (3) We add slice-wise radial strains to the previously computed circumferential and longitudinal strains. Radial strains are often challenging to estimate and the reported values widely vary in the literature as a function of acquisition sequence and method employed for their calculation (Wang et al. 2021).

The subsequent parts of the paper are organized as follows: The methods employed to compute cardiac strains from DENSE and Cine MRI data, the data acquisition, and the statistical analysis are described in Sects. 2.1–2.5. The computational deforming phantom used to test the presented methodology is described in Sect. 2.3 and the test results are shown in Sect. 3.1. Group-wise and samples of subject-wise strains are shown and commented in Sect. 3.2. The remaining subject-wise plots comparing strains computed from the two-slice DENSE model and one-slice Cine model are reported in Appendix A. A sensitivity analysis to image segmentation of the one-slice Cine model is presented in Sect. 3.3. Section 3.4 and Sect. 4 summarize, respectively, the limitations and the main findings of this study.

## Methodology

**Fig. 1:**
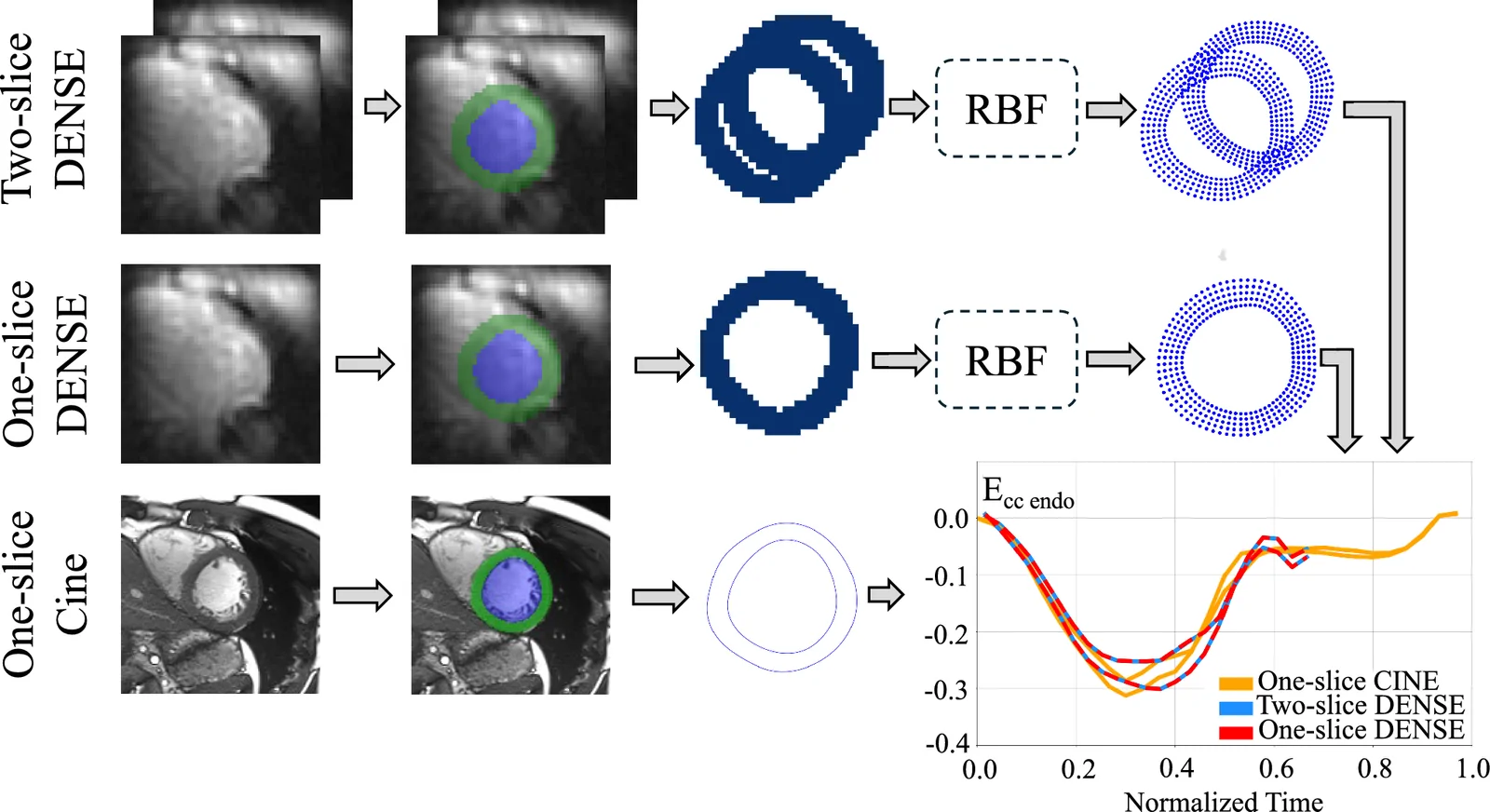
Overview of the DENSE and Cine models. The two-slice DENSE model (top) relies on two short-axis DENSE images, which are automatically segmented to isolate the left ventricle myocardial (LVM) voxels. After processing the DENSE phase data in the LVM, the voxel-wise displacements are interpolated using radial basis functions (RBFs) and strains are computed at the location of interest. The same procedure is followed by the one-slice DENSE model (middle), except for longitudinal strains that are estimated as in the one-slice Cine model based on the LVM mask area. Strain estimation in a one-slice Cine model (bottom) relies on a single slice LVM segmentation from which mask area and epicardial/endocardial contours are computed

The overall implementation of the DENSE- and Cine-based models is presented schematically in Fig. 1. The DENSE images are first segmented using an nnU-Net (Isensee et al. 2021) model. The mask predicted by the nnU-Net model is employed to isolate the myocardial voxels, from which displacements are computed using the DENSE-Analysis toolbox (Spottiswoode et al. 2007; Gilliam and Suever 2021). Discrete voxel-wise displacement data are transformed in a continuous displacement field using a radial basis function interpolation, from which strains are finally computed.

The Cine model employs a separate nnU-Net (Isensee et al. 2021) model to isolate the left ventricle myocardium (LVM), from which the epicardial and endocardial contours are identified. These contours and area of the segmented mask are then used to approximate slice-wise circumferential, radial, and longitudinal strains.

The DENSE- and Cine-based models are presented in details next, followed by the computational phantom and the acquired volunteer dataset used to test and apply the presented models. The code for each model is freely available at https://github.com/CBL-UCF/SingleSliceCardiacStrains.

### Strain calculations from DENSE images

In this study, two separate models have been defined using DENSE MRI short-axis imaging data: a two-slice model and a one-slice model based, respectively, on two and one short-axis DENSE images. Strains computed from the two-slice model are based on the full 3D displacement field, including displacement gradients along the longitudinal axis. In contrast, strains computed from the one-slice model are based on the full 3D displacement field confined to one short-axis slice, without knowledge of the displacement variation along the longitudinal axis. Although the former provides more complete information, the latter requires half the data and therefore decreases the acquisition time. The methods employed to process the DENSE short-axis data, extract voxel-wise displacements, and compute cardiac strains are described next.

#### DENSE image segmentation

To automate the identification of the left ventricle myocardium (LVM) and cavity (LVC), we utilize the nnU-Net (Isensee et al. 2021) framework, a self-configuring DL architecture specifically designed to segment medical images. The main advantage of this framework is its ability to automatically optimize the hyper-parameters of the DL architecture, such as intensity normalization and resampling, alongside critical training parameters such as patch size and batch size. Our implementation employs the 3D full-resolution nnU-Net configuration (in our case 2D + time) allowing the model to leverage temporal continuity throughout the cardiac cycle. This helps the DL model to detect the accurate boundaries of the LV during the cardiac cycle.

Each input slice was represented as a four-channel image containing magnitude and the *X*, *Y*, *Z* phase components, the latter encoding displacement information. By utilizing both magnitude and phase information, the model achieves better performance than models trained on DENSE magnitude only, particularly in early or late cardiac phases where image contrast is typically low (Jahromi et al. 2024).

The model was developed using a fivefold cross-validation strategy on a dataset comprised of twenty (N=20) volunteers, partitioned into 80% for training and 20% for validation. This dataset included 130 short-axis slices—covering the left ventricle from apex to base—and 3789 cardiac frames. To generate reference ground-truth masks necessary to train the DENSE nnU-Net model, a DENSE manual segmentator was created in Python and made publicly available at https://github.com/CBL-UCF/DENSEmanualSegmentator.

The optimization utilized a combined DICE and cross-entropy loss function where training is performed for up to 1000 epochs. For the final inference, the best-performing epoch weight of each fold is utilized in an ensemble strategy that averages voxel-wise softmax probabilities across all folds.

In rare frames where the network failed to generate closed or valid epi- and endocardial contours (<0.2% of all frames), a post-processing step was applied to maintain temporal consistency. Missing or invalid frames were corrected through linear interpolation or extrapolation from neighboring valid frames across the cardiac cycle, providing anatomically acceptable masks for the subsequent displacement and strain analyses.

#### Strain calculations in one- and two-slice DENSE models

Once the segmentation mask is predicted using the trained nnU-Net model, the DENSE-Analysis toolbox (Spottiswoode et al. 2007; Gilliam and Suever 2021) was adopted for displacement calculation. To do so, the endo- and epicardial contours for each cardiac frame were extracted as splines based on the segmented mask and inserted into the DENSE-Analysis toolbox without relying on a manual or motion guided segmentation. (The latter would still require an initial manual segmentation and further manual adjustments.) Based on the splines from the nnU-Net model predictions, the DENSE-Analysis toolbox initiated the post-processing tasks, including spatiotemporal phase unwrapping and the tracking of tissue voxel trajectories. This integration of nnU-Net and DENSE-Analysis toolbox significantly reduces processing time and mitigates the potential bias introduced by the user carrying out the segmentation.

Once the voxel-wise Lagrangian displacements have been computed by the DENSE-Analysis toolbox, a cubic radial basis function (RBF) including a linear polynomial is employed to interpolate the displacement data and transform it to a continuous and differentiable field $\textbf{U}(\textbf{X})$. (In our implementation, we have used the SciPy library by Virtanen et al. 2020.) $\textbf{X}$ denotes the reference configuration, which, in this work, corresponds to the beginning of systole. For each displacement component $\text {U}_i(\textbf{X})$, the 3D RBF-based interpolation is defined as:

1 $$\begin{aligned} \text {U}_i(\textbf{X}) = \sum _{j=1}^{N_D} w_j \, \Phi \!\left( \Vert \textbf{X}-\textbf{X}_j\Vert \right) + a_0 + a_x X + a_y Y + a_z Z \, , \end{aligned}$$

where $\Phi (r)=r^3$ is the cubic radial basis kernel, $\textbf{X}_j$ are the sampled voxel locations ($j\in [1,N_D]$, where $N_D$ is the number of DENSE voxels in the LVM), $w_j$ are the RBF coefficients, and $(a_0 + a_x X + a_y Y + a_z Z)$ is the linear polynomial added to the cubic kernel. Before constructing the RBF-based interpolation, each DENSE slice is rigidly translated in the short-axis (*X*–*Y*) plane so that its barycenter (i.e., the LVM barycenter) is positioned at the origin. In the two-slice DENSE model, this rigid registration in the reference configuration guarantees that both slices’ barycenters are aligned along the *Z*-axis and mitigates slice misalignment due, for example, to different breath-holds during data acquisition.

Based on $\textbf{U}(\textbf{X})$, the deformation gradient tensor $\textbf{F} = \nabla _{\textbf{X}}\textbf{U} + \textbf{I}$ is evaluated to then compute the Green–Lagrange strain tensor $\textbf{E} = \frac{1}{2}(\textbf{F}^{\text {T}}\textbf{F}-\textbf{I})$ at any point of interest in the myocardium. Circumferential ($\text {E}_{\text {cc}}$), longitudinal ($\text {E}_{\text {ll}}$), and radial ($\text {E}_{\text {rr}})$ strains are then computed as $\text {E}_{\text {nn}} = \textbf{n}\cdot \textbf{E}\textbf{n}$, where the unit vector $\textbf{n}$ is selected along the circumferential, longitudinal, or radial direction in the reference configuration in function of the strain component to be computed. The radial direction $\textbf{r}$ is selected as the vector pointing from the barycenter of the myocardium to the point of interest and the circumferential direction $\textbf{c}$ is computed as perpendicular to $\textbf{r}$ in the short-axis slice (*X*–*Y*) plane. The longitudinal direction $\textbf{l}$ is selected as perpendicular to the short-axis slice plane (in the Z direction).

The two-slice DENSE model adopted in this work follows this procedure to compute cardiac strains as the full deformation gradient tensor $\textbf{F}$ can be computed from the displacement information on two consecutive short-axis slices. In the one-slice DENSE model, the displacement components are interpolated with a 2D RBF interpolator. In this case, only the components $\text {F}_{\text {i}\Gamma }$ (Γ=1,2; i=1,2,3) of $\textbf{F}$ can be computed as components $\text {F}_{\text {i3}}$ (i=1,2,3) cannot be evaluated from single slice displacement information. The partial deformation gradient tensor computed from single slice displacements still allows to estimate $\text {E}_{\text {cc}}$ and $\text {E}_{\text {rr}}$ as in the two-slice DENSE model. However, $\text {E}_{\text {ll}}$ cannot be computed from $\textbf{F}$ and is approximated using Eq. 5 as in the one-slice Cine model.

Finally, to compare the strains computed using the DENSE and Cine models across volunteers, both the Cine and DENSE image acquisition time are normalized to the [0, 1) interval based on the RR interval recorded during the Cine MRI data acquisition. Specifically, the normalized Cine time $t_{\text {C}}$ is computed as:

2 $$\begin{aligned} t_{\text {C}} = (\text {n}_{\text {C}}-1)/\text {N}_{\text {C}} \, , \end{aligned}$$

where $\text {n}_{\text {C}}$ is the Cine frame number (starting from 1) and $\text {N}_{\text {C}}$ is the total number of acquired Cine images. Similarly, the normalized DENSE time $t_{\text {D}}$ is computed as:

3 $$\begin{aligned} t_{\text {D}} = \frac{t_{1} + (\text {n}_{\text {D}}-1)\Delta t_{\text {D}}}{\text {RR}} \, , \end{aligned}$$

where $t_{1}$ is the time at which the first DENSE image is acquired due to the encoding process ($t_{1}\approx 17.12$ ms in our protocol), $\text {n}_{\text {D}}$ is the DENSE frame number (starting from 1), and $\Delta t_{\text {D}}$ is the time interval between acquired DENSE images (TR). We note that, since the DENSE acquisition does not generally cover the full cardiac cycle, DENSE-derived strains are not available in the entire [0, 1) interval. The normalized Cine and DENSE times allow to align strains computed across subjects and sequences. However, inconsistencies in the temporal registration may still exist due to: (1) heart rate variability between image acquisitions in the same subject; and/or (2) heart rate differences across subjects and the fact that different phases of the cardiac cycle do not scale uniformly as a function of heart rate. Although possible, we expect these sources of temporal misalignment to be small.

### Strain calculations from Cine images

The proposed model to compute cardiac strains from Cine MR images is based on single slice data. The LVM mask is first segmented and epicardial/endocardial contours are extracted to estimate slice-wise circumferential and radial strains. Longitudinal strains are estimated based on the LVM mask area directly.

#### Cine image segmentation

An nnU-Net model (Isensee et al. 2021) is separately trained to segment short-axis Cine images. The model training was based on approximately 1200 images from 152 short-axis slices belonging to twenty different volunteers. Images corresponding to short-axis slices acquired from apex to base were manually segmented either at every frame, every other frame, or only at beginning and end systole. The model was trained using a fivefold cross-validation approach, in which each fold consisted of sixteen subjects in the training set and four subjects in the validation set with a balanced number of images across both sets. The ground-truth masks necessary to train the Cine nnU-Net model were created in 3D Slicer (Fedorov et al. 2012, 2026).

The choice of using an nnU-Net model versus the UNet$_\text {AG}$ model (Von Zuben et al. 2023) used in our previous work (Marques et al. 2025) was made to maintain the same model architecture for both DENSE and Cine image segmentation and due to the wide use of the nnU-Net architecture in the literature.

#### Strain calculations in the one-slice Cine model

As no point-wise displacement information is directly available in the one-slice Cine model, strains are approximated based on epicardial and endocardial contour motion. First, each frame is segmented using the trained nnU-Net model. Subsequently, the voxels at the LVM boundaries are identified with the help of the Skimage (Van der Walt et al. 2014) Python library. The epicardial and endocardial voxels are then fitted with two separate splines defined in polar coordinates (r,θ) based on the least square method. The origin of the polar coordinate system for each slice is defined as the LVM barycenter, which is computed from the LVM mask at every time frame. This step eliminates the effect of rigid translations between frames and registers the origins of the reference system from which *r* and θ are measured. (θ is measured from the horizontal axis.) Each spline is based on sixteen control points, approximately equally spaced in θ, the polar coordinate in the circumferential direction. The created spline functions are then used to find the radii r(θ) for the epicardial and endocardial contours. Based on the epicardial and endocardial splines and the mask of the segmented LVM, we then estimate slice-wise strains. Circumferential and longitudinal strains are estimated according to our previous work (Marques et al. 2025), while the estimation of radial strains is newly added in this study.

Epicardial and endocardial contour circumferential strains $\text {E}_{\text {cc}}$ are computed as:

4 $$\begin{aligned} \text {E}_{\text {cc}}(t) = \frac{1}{2}\left[ \left( \frac{p(t)}{p_{\text {ref}}}\right) ^2 - 1 \right] \, , \end{aligned}$$

where *p*(*t*) and $p_{\text {ref}}$ are, respectively, the length of the splines tracing the LVM boundaries at time *t* and in the reference configuration. (As for the DENSE models, the reference configuration is defined at the beginning of systole and corresponds to the first Cine image acquired with the ECG-gated protocol.) $\frac{p(t)}{p_{\text {ref}}}$ represents an approximation for the stretch ratio in the circumferential direction. At each time frame, epicardial and endocardial splines are used to compute $\text {E}_{\text {cc}}$ at the epicardium ($\text {E}_{\text {cc,epi}}$) and endocardium ($\text {E}_{\text {cc,endo}}$), respectively. (One epicardial value and one endocardial value are computed per time frame.) The spline length is computed integrating the differential arc length as $p=\int ^{2\pi }_0\sqrt{r(\theta )^2+\left( \frac{\textrm{d}r}{\textrm{d}\theta }\right) ^2}\textrm{d}\theta$  (Larson and Edwards 2009).

Slice-wise longitudinal strain $\text {E}_{\text {ll}}$ is computed assuming that the longitudinal shortening is constant across the short-axis slice and that the LVM volume for every slice is conserved. The latter approximation is based on the assumption of myocardium low tissue compressibility (e.g., Rodriguez et al. 2006). Under these assumptions, the myocardium volume can be approximated as $V=h_{\text {ref}}A_{\text {ref}}=h(t)A(t)$, where *h* and *A* are the slice height and area in the reference and time *t* configurations. Accordingly, the stretch ratio $h(t)/h_{\text {ref}}$ in the longitudinal direction can be estimated as $h(t)/h_{\text {ref}} = A_{\text {ref}}/A(t)$ and $\text {E}_{\text {ll}}$ can be estimated as:

5 $$\begin{aligned} \text {E}_{\text {ll}}(t) = \frac{1}{2}\left[ \left( \frac{A_{\text {ref}}}{A(t)}\right) ^2 - 1 \right] \, . \end{aligned}$$

The areas $A_{\text {ref}}$ and *A*(*t*) in the reference and time *t* configurations are computed as the area of the LVM mask. Although the area values can be computed as the area of the region in between the endocardial and epicardial splines, in this work we evaluate *A*(*t*) directly as the sum of all the pixels in the LVM mask. One $\text {E}_{\text {ll}}$ value is obtained per time frame per slice.

Finally, mid-wall radial strains $\text {E}_{\text {rr}}$ are approximated as:

6 $$\begin{aligned} \text {E}_{\text {rr}}(t,\theta ) = \frac{1}{2}\left[ \left( \frac{w(t,\theta )}{w_{\text {ref}}(\theta )}\right) ^2 - 1 \right] \, , \end{aligned}$$

where $w_{\text {ref}}(\theta )$ and w(t,θ) denote the myocardial wall thickness in the reference and time *t* configurations at the location identified by the polar coordinate θ. The wall thickness *w* is computed as the distance between the endocardial and epicardial splines in the radial direction measured from the LVM barycenter, i.e., $w(t,\theta ) = r_\text {epi}(t,\theta ) - r_\text {endo}(t, \theta )$. $\text {E}_{\text {rr}}(t,\theta )$ is computed at 360 θ locations ($1^{\circ }$ apart) and the median value is reported as the slice-wise radial strain.

Both circumferential (Eq. 4) and radial (Eq. 6) strain approximations are based on the epicardial and endocardial contours computed in the same short-axis plane throughout the cardiac cycle. A consequence of this approximation is that out-of-plane motion and torsion are neglected, leading to potential biases in the computed strain values.

### Validation using the phantom model

To test the proposed strain calculations in both DENSE- and Cine-based models, we adopt the computational phantom described in Verzhbinsky et al. (2019) and Perotti et al. (2021). This phantom is a thick-walled hollow cylinder (in this study we set the inner and outer radii equal to 25 mm and 35 mm, respectively) that simulates the deformation of a mid-ventricular, left ventricular section using a polynomial deformation mapping. The phantom allows to compare the strains computed with the proposed models to ground-truth values at any location within its domain. The prescribed deformation mapping allows to simulate wall thickening (using a quadratic polynomial in the radial direction), a uniform longitudinal shortening (using a linear polynomial in the longitudinal direction), and slice-wise twist (prescribing circumferential displacement that vary linearly in the longitudinal direction) during contraction. Virtual DENSE images are generated at longitudinal locations that are 8 mm apart (Z=8 mm and Z=16 mm) and with an in-plane spatial resolution of 2.5×2.5 mm$^2$ to replicate the DENSE image acquisition protocol used in the majority of this study.

The DENSE images (magnitude and phase) corresponding to the phantom motion are generated at beginning and peak systole and are used to test the two- and one-slice DENSE models.

The phantom magnitude images (at Z=16 mm) are also used to test the calculations carried out in the one-slice Cine model and based only on the endocardial/epicardial contours and LVM mask. To account for the higher spatial resolution typical of Cine images, the phantom images are generated doubling the in-plane spatial resolution to 1.25×1.25 mm$^2$. Subsequently, for testing the one-slice Cine model, image segmentation is carried out based on the signal intensity of the virtual DENSE magnitude images used as an approximation for Cine images, i.e., voxels with intensity greater than or equal to 0.5 are considered myocardium. (The signal intensity is normalized between 0 and 1.) The obtained mask is finally passed to the one-slice Cine model pipeline to compute strains and compare with the computational phantom values. To test the one-slice Cine model, we used endocardial and epicardial contours obtained from: (1) the exact phantom formulation (no mask is generated in this case); (2) the mask generated from magnitude images obtained with signal-to-noise ratio (SNR) equal to 1000; and (3) the mask generated from magnitude images obtained with SNR = 20. Ten phantom simulations for both SNR equal to 1000 and SNR equal to 20 are generated to evaluate the one-slice Cine model.

### Data acquisition

The imaging data for this study were collected in forty (N=40) healthy volunteers (38.75 years ± 12.9 years old, 19 males and 21 females) under an Institutional Review Board (IRB) approved protocol (Stanford Administrative Panel on Human Subjects in Medical Research, reference numbers FWA00000929 and FWA00000934) with all participants providing written informed consent. Anonymized data were received and analyzed at the University of Central Florida under IRB STUDY00004491. The data are available as part of the ‘Human MRI Data to Quantify Cardiac Performance’ repository at https://purl.stanford.edu/xt223hz2438

The DENSE images were acquired using an ECG-gated protocol with breath-holds. While the majority of images were obtained with 2.5×2.5 mm$^2$ in-plane spatial resolution, a small subset of images was acquired with a higher in-plane resolution of 2×2 mm$^2$. A standard slice thickness of 8 mm was maintained across the entire cohort. (A slice thickness equal to 8 mm was chosen as a compromise between spatial resolution and signal-to-noise ratio.) Specific DENSE sequence parameters included: repetition time (TR) of 15 ms with view sharing and 30 ms without view sharing, echo time (TE) of 1.08 ms, FOV = 200×200, $15^{\circ }$ flip angle, a simple 4-point displacement encoding strategy, in-plane and out-of-plane displacement encoding frequency ($k_{e}$) of 0.10 cycles/mm and 0.08 cycles/mm, respectively.

The cardiac bSSFP Cine images were also acquired using an ECG-gated protocol with breath-holds. The Cine image spatial resolution was $1.17 \times 1.17 \times 8$ mm$^3$ and specific sequence parameters were: repetition time (TR) of 32 ms, echo time (TE) of 1.37 ms, FOV = 253×300, acceleration = GRAPPA × 3. The average RR interval recorded during Cine data acquisition across all volunteers was 996 ms ± 136 ms.

For each subject, the same two mid-ventricular short-axis slices adjacent to each other (0% slice gap) were selected in both the DENSE and Cine acquisitions to compute cardiac strains.

### Statistical analysis

Myocardial strain components (peak median systolic $\text {E}_{\text {cc,epi}}$, $\text {E}_{\text {cc,endo}}$, $\text {E}_{\text {ll}}$, and $\text {E}_{\text {rr,mid}}$) were compared across the three models: two-slice DENSE, one-slice DENSE, and one-slice Cine. The normality of the dataset was evaluated using the Shapiro–Wilk test. For normally distributed strain components, a one-way repeated-measures ANOVA was performed. For cases where the sphericity assumption was violated, Greenhouse–Geisser corrections were applied to *p*-values (Field 2009). Post hoc head-to-head comparison was made using paired t-test with Bonferroni correction for multiple comparisons.

For components with non-normal distribution, the nonparametric Friedman test was used. Post hoc head-to-head comparison was made using Wilcoxon signed-rank tests with Bonferroni correction for multiple comparisons. The systematic difference between models was evaluated using Cohen’s *d* effect size (Cohen 2013).

The two one-sided tests (TOST) is performed to evaluate the equivalence between selected strain measures obtained with different models (Schuirmann 1987).

In this study, we consider a significance level α=0.05. All statistical analyses were performed using the Pingouin library (Vallat 2018) in Python 3.12. Peak median systolic strains computed per each slice using the three models are also compared using pair-wise differences. Median and first (Q1) and third (Q3) quartiles of pair-wise strain differences are reported.

## Results and discussion

The proposed models are evaluated using both a computational phantom and data acquired in healthy volunteers. The computational phantom allows to evaluate the models against a known ground-truth, although this comparison is limited to an idealized cylindrical geometry and simple motion. In contrast, the volunteer dataset allows to evaluate the proposed models in anatomical geometries and using physiological motion. In this section both evaluations are discussed in detail.

**Fig. 2:**
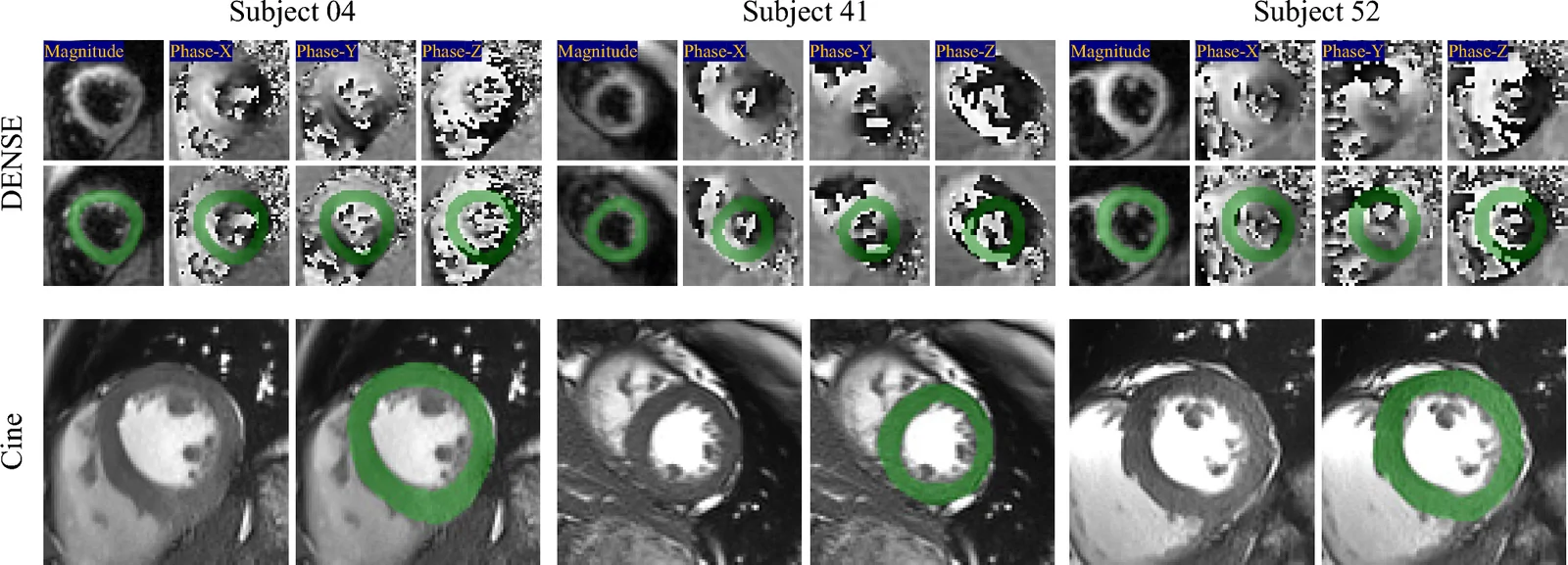
Representative DENSE (top) and Cine (bottom) images with corresponding segmentations of the left ventricle myocardium (in green) obtained using the trained DENSE and Cine nnU-Net models. The input DENSE images include magnitude and phase (in *X*, *Y*, and *Z* directions) data, while the input Cine images include only magnitude data. All images shown in this figures were not seen by the nnU-Net models during the training and validation phases

As the initial step in both the DENSE and Cine models consists in identifying the left ventricle myocardium (LVM), before evaluating the models using the computational phantom and volunteer data, we first briefly evaluate the nnU-Net segmentation models. Figure 2 illustrates the LVM segmentations obtained with the trained nnU-Net models, showing the successful delineation of the LVM mask in representative cases. Overall, LVM Dice similarity coefficient obtained on the validation sets averaged over the fivefold cross-validation is ≈0.89 and ≈0.90 for the DENSE and Cine segmentation models, respectively.

### Phantom validation

The two-slice and one-slice DENSE models are evaluated considering two scenarios: (1) In the first scenario, the nnU-Net model is used to segment the DENSE images generated from the phantom and the analytical phantom displacements are assigned to the image voxels, i.e., the voxel displacements are exact. This scenario is used to test the effect of image segmentation and displacement interpolation. (2) In the second scenario, the nnU-Net model is used for segmentation and the voxel displacements are computed using the DENSE-Analysis toolbox, i.e., the full pipeline is tested using the DENSE images generated from the computational phantom. In both scenarios, the LVM is divided transmurally in five equal layers and strains are computed in each layer at 72 equally spaced ($5^\circ$ apart) points in the circumferential direction. This strategy allows to highlight regions where strain errors may localize. All results presented for the two-slice and one-slice DENSE models are obtained using a signal-to-noise ratio equal to twenty (SNR = 20) in the computational phantom.

**Fig. 3:**
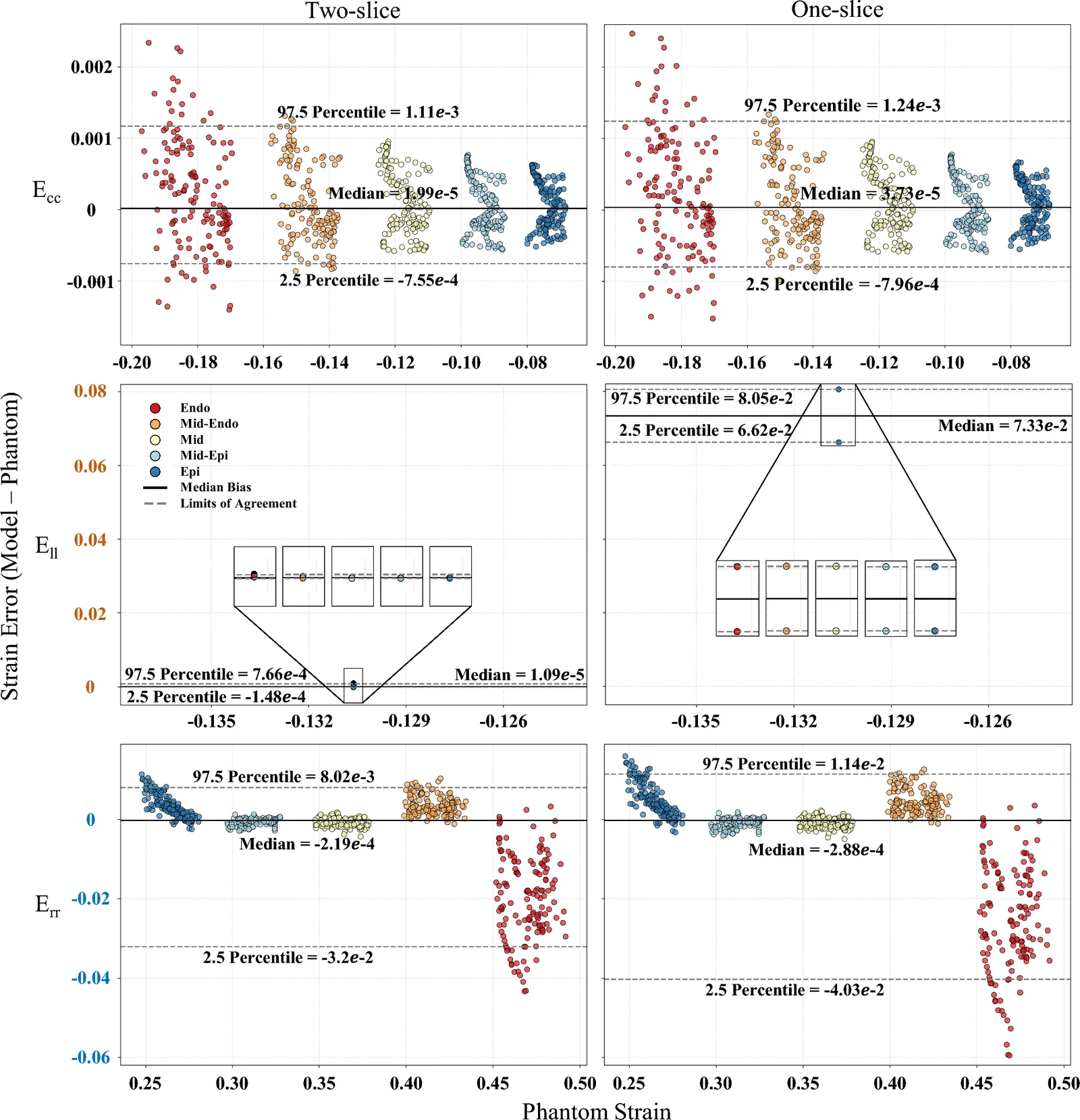
Case 1: nnU-Net (applied to images obtained with SNR = 20) + exact (analytical) displacements – Strain errors versus ground-truth strains. Strain errors are computed by comparing ground-truth phantom strains with the ones obtained using the two-slice and one-slice DENSE models in the first test scenario. In this case, the DENSE models’ strains are computed using the nnU-Net segmentation followed by assigning exact analytical displacements at the myocardial voxels within the LVM mask. This test isolates the effect of nnU-Net segmentation and RBF interpolation on the computed strains. Circumferential ($\text {E}_{\text {cc}}$), longitudinal ($\text {E}_{\text {ll}}$), and radial ($\text {E}_{\text {rr}}$) strains errors versus ground-truth phantom strains at peak systole are presented for the two-slice (left) and one-slice (right) models. Strain markers are colored based on transmural myocardial segments: endocardium (red), mid-endocardium (orange), mid-myocardium (yellow), mid-epicardium (cyan), and epicardium (blue). The solid black line defines the aggregate median bias of error across all segments, while the dashed gray lines represent the limits of agreement (2.5 and 97.5 percentiles)

**Case 1: nnU-Net + exact displacements: ** Figure 3 presents the validation results obtained when exact analytical displacements are assigned to the voxels in the mask predicted by the nnU-Net model. In this case, we observe that:

- Circumferential strains $\text {E}_{\text {cc}}$ are computed with high accuracy, with very small median and 2.5 and 97.5 percentiles error (in magnitude $\lessapprox 0.1\%$) for both two-slice and one-slice models at all transmural locations.
- Longitudinal strains $\text {E}_{\text {ll}}$ are computed with high accuracy by the two-slice DENSE model, leading to near zero error. However, $\text {E}_{\text {ll}}$ computed using the one-slice DENSE model is inaccurate presenting a median error equal to $\approx 7.3\%$. Since in the one-slice DENSE model $\text {E}_{\text {ll}}$ is computed based only on the LVM area, having the analytical phantom displacements does not affect the $\text {E}_{\text {ll}}$ calculation, which remains highly sensitive to the LVM segmentation.
- Radial strains $\text {E}_{\text {rr}}$ are computed with good accuracy (strain error $\lessapprox 2\%$) in all transmural layers except in the endocardial layer where the strain error reaches (in magnitude) $\approx 4\%$ and $\approx 6\%$ in the two-slice and one-slice models, respectively. This larger error in the endocardial region may be due to a high sensitivity of $\text {E}_{\text {rr}}$ to segmentation errors.

**Fig. 4:**
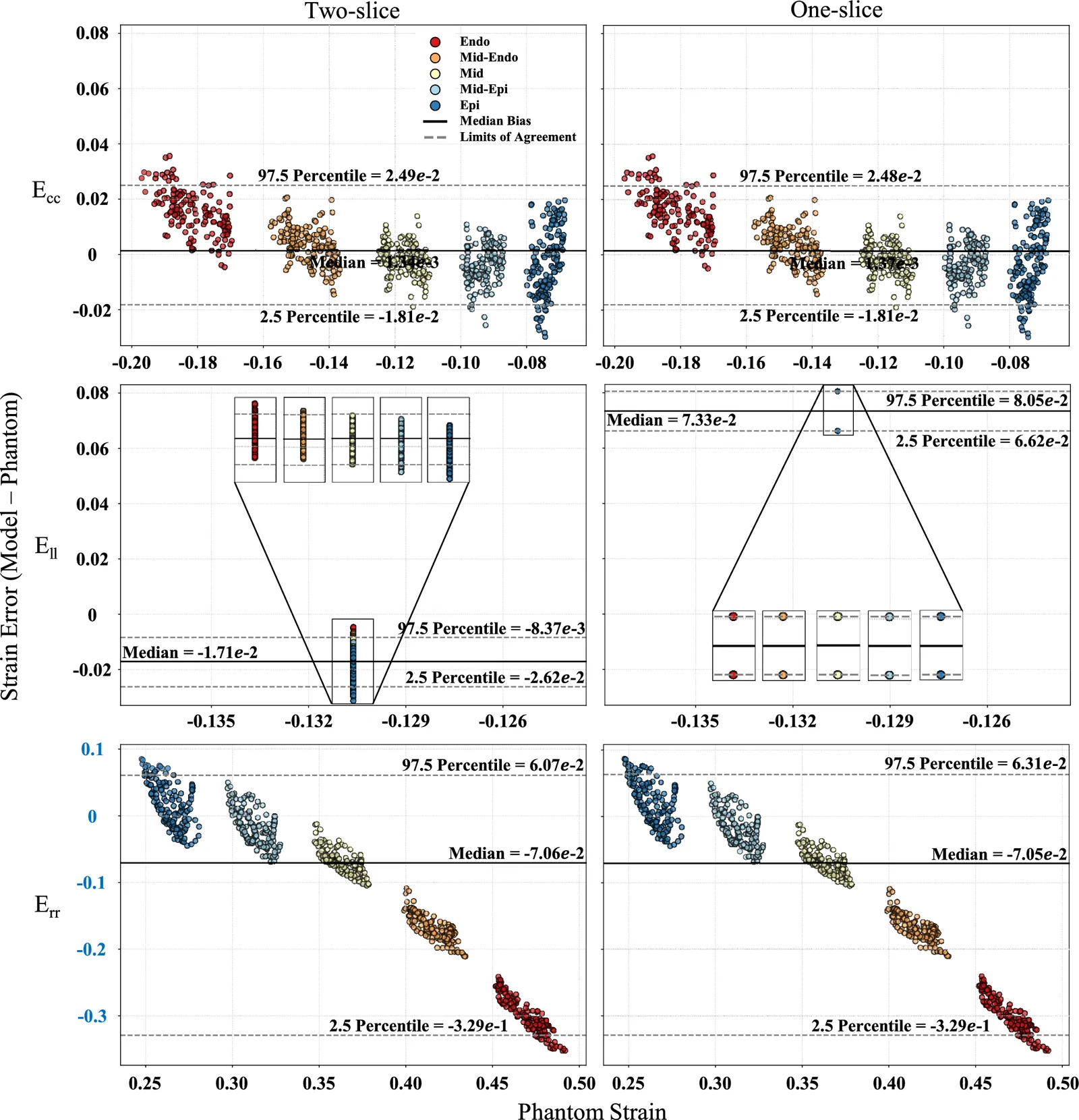
Case 2: nnU-Net + computed displacements (testing the full automated pipeline) – Strain errors versus ground-truth strains shown for one representative case using phantom generated images with SNR = 20. Strain errors are computed by comparing ground-truth phantom strains with the ones obtained using two-slice and one-slice DENSE models in the second test scenario. In this case, the DENSE models’ strains are computed using the full automated pipeline, i.e., nnU-Net segmentation followed by DENSE-Analysis toolbox displacement calculation. Circumferential ($\text {E}_{\text {cc}}$), longitudinal ($\text {E}_{\text {ll}}$), and radial ($\text {E}_{\text {rr}}$) strain errors versus ground-truth phantom strains at peak systole are presented for the two-slice (left) and one-slice (right) models. Strain markers are colored based on transmural myocardial segments: endocardium (red), mid-endocardium (orange), mid-myocardium (yellow), mid-epicardium (cyan), and epicardium (blue). The solid black line defines the aggregate median bias of error across all segments, while the dashed gray lines represent the limits of agreement (2.5 and 97.5 percentiles)

**Case 2: nnU-Net + computed displacements: ** Figure 4 presents the validation results obtained using the full DENSE model pipeline, i.e., the nnU-Net predicted segmentation is combined with the DENSE-Analysis toolbox for displacement calculation. In this case, we observe that:

- Circumferential strains $\text {E}_{\text {cc}}$ are computed with good accuracy (median error $\approx 0.1\%$) as in the previous test scenario, although the 2.5–97.5 percentiles range is wider for both the two-slice and one-slice models ($\approx [-1.8\%, 2.5\%]$) versus the 2.5–97.5 percentiles range computed in the first test scenario ($\approx [-0.08\%, 0.12\%]$).
- Longitudinal strains $\text {E}_{\text {ll}}$ are still computed with reasonable accuracy using the two-slice DENSE model (median error $\approx -1.7\%$ and 2.5–97.5 percentiles range $\approx [-2.6\%, -0.8\%]$). As in the first test scenario, $\text {E}_{\text {ll}}$ computed using the one-slice DENSE model is inaccurate and presents the same median error ($\approx 7.3\%$) since its calculation depends exclusively on the area of the LVM segmented mask.
- Radial strains $\text {E}_{\text {rr}}$ computed with the DENSE models present significant errors in this case (median $\approx -7.1\%$ and 2.5–97.5 percentiles range $\approx [-33\%, 6\%]$), with larger discrepancies toward the endocardium. This large error in radial strains shows the sensitivity of $\text {E}_{\text {rr}}$ to segmentation errors and imaging noise. This error may be reduced by improving the processing of the displacement field derived from the segmented LVM voxels.

**Fig. 5:**
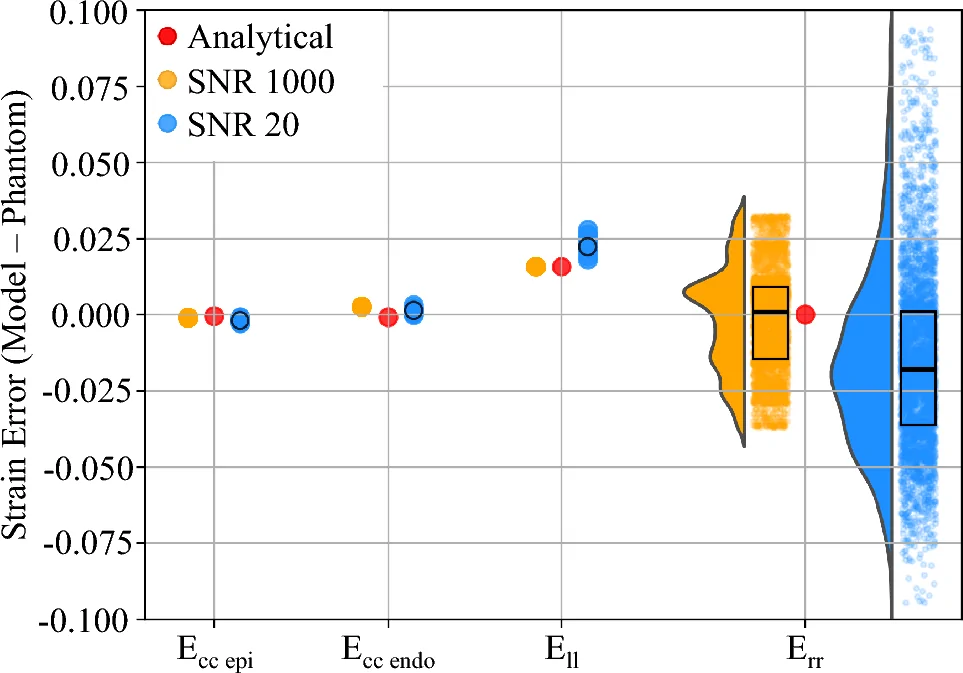
One-slice Cine model versus computational phantom – Strain errors are shown for models based on: exact epicardial and endocardial contours (red), and contours based on LVM masks generated from SNR 1000 (orange) and SNR 20 (blue) images. Results are shown based on 10 phantom simulations to represent noise variability. For SNR=20, the blue circles with the black outlines represent the median strain error. Endocardial and epicardial circumferential strains $\text {E}_{\text {cc}}$ values approximately match the ground-truth values (absolute errors less than 0.1%) if exact contours are employed, and small errors ($\lessapprox 0.5\%$ in magnitude) also arise using the contours generated from the LVM masks. Longitudinal strain $\text {E}_{\text {ll}}$ presents an error of 1.58% even if computed based on the analytical contours. The median error in $\text {E}_{\text {ll}}$ is 1.57% and 2.23% when computed from the LVM masks generated with SNR=1000 and SNR=20, respectively. This error is due to the compressibility of the myocardium that, even if low, is included in the computational phantom and neglected in the one-slice Cine model. Radial strain $\text {E}_{\text {rr}}$ computed using the one-slice Cine model matches the mid-wall analytical strain value when exact contours are used. Depending on the location, larger strain errors appear when $\text {E}_{\text {rr}}$ is computed from the contours generated from the LVM masks. The box on the right of each raincloud plot represents the IQR, while the thick line represents the median computed using 360 points equally spaced in the circumferential direction per each phantom simulation

The same computational phantom used to test the DENSE models was employed to test the one-slice Cine model. Exact (analytical) epicardial and endocardial contours, and contours generated from SNR = 1000 and SNR = 20 images are used to test the model in perfect conditions (analytical contours) and when the LVM boundaries are generated from masks segmented in the presence of noise (SNR = 20) or with virtually no noise (SNR = 1000). To account for variability in segmentation due to the simulated noise, the phantom was simulated ten times for each SNR and the median [Q1, Q3] results are reported (since for SNR = 1000, there is virtually no variability in the $\text {E}_{\text {cc}}$ and $\text {E}_{\text {ll}}$ results, only the median values are reported for SNR = 1000). Based on the results presented in Fig. 5, we observe that:

- Contour endocardial and epicardial circumferential strains $\text {E}_{\text {cc}}$ approximately match ground-truth values when computed based on the exact endocardial and epicardial contours (absolute errors less than 0.1%). A small error is also present when endocardial and epicardial $\text {E}_{\text {cc}}$ are computed from the LVM masks obtained with SNR = 1000 and SNR = 20: Median $\text {E}_{\text {cc,epi}}$ error is equal to -0.10% and -0.19% [-0.24%, -0.18%] for SNR = 1000 and SNR = 20, respectively, while median $\text {E}_{\text {cc,endo}}$ error is equal to 0.27% and 0.14% [0.06%, 0.18%] for SNR = 1000 and SNR = 20, respectively.
- Slice-wise longitudinal strain $\text {E}_{\text {ll}}$ presented an error even when computed from exact endocardial and epicardial contours. This is due to neglecting the myocardium compressibility that, although small, is included in the computational phantom. By neglecting the small volume reduction included in the computational phantom, the one-slice Cine model underestimates in magnitude $\text {E}_{\text {ll}}$. (This corresponds to a positive error equal to 1.58%.) The error in $\text {E}_{\text {ll}}$ remains practically constant or increases slightly when the segmentation from the SNR=1000 and SNR=20 images are considered: Median $\text {E}_{\text {ll}}$ error is equal to 1.57% and 2.23% [2.13%, 2.53%] for SNR = 1000 and SNR = 20, respectively.
- Slice-wise radial strain $\text {E}_{\text {rr}}$ matches the ground-truth mid-wall radial strain value when the exact endocardial and epicardial contours are employed. When $\text {E}_{\text {rr}}$ is computed using the contours of the LVM masks generated with SNR=1000 and SNR=20, a larger error variability is present: The median errors are equal to 0.08%[-1.48%, 0.90%] (2.5–97.5 percentiles range = 6.40%) and -1.81% [-3.62%, 0.09%] (2.5–97.5 percentiles range = 12.47%)for SNR = 1000 and SNR = 20, respectively. The larger strain errors are due to the approximation of the endocardial and epicardial contours and the sensitivity of $\text {E}_{\text {rr}}$ to errors in wall thickness in the reference and deformed configurations according to Eq. 6.

In all cases generated with the considered phantom, the median strain errors computed using SNR = 1000 and SNR = 20 images are relatively small, with the relatively larger errors for $\text {E}_{\text {ll}}$ and $\text {E}_{\text {rr}}$ computed using SNR = 20. The main difference consists in the larger variability in strain errors computed using SNR=20 due to, as expected, larger variations in image segmentations.

### Results in healthy volunteers

**Fig. 6:**
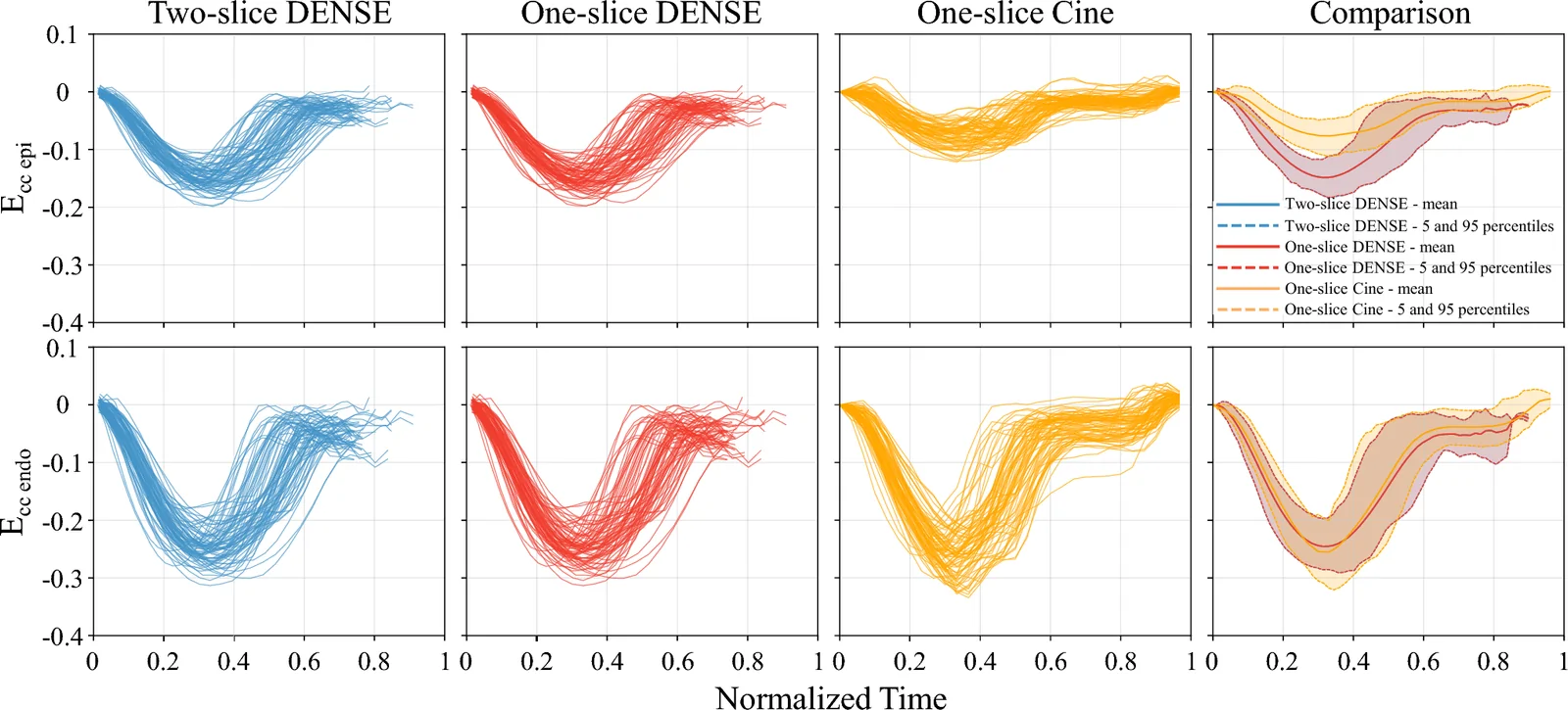
Epicardial (top) and endocardial (bottom) slice-wise circumferential $\text {E}_{\text {cc}}$ strains (N=40 subjects) versus normalized time. Each line in the first three columns represents the circumferential strain per slice obtained (from left to right) with the two-slice DENSE model, one-slice DENSE model, and one-slice Cine model. Median strain values are reported for the DENSE models as multiple $\text {E}_{\text {cc}}$ values per slice are computed. The mean of all $\text {E}_{\text {cc}}$ curves per model are reported in the right column as solid lines. (Shaded regions represent the range between the 5 and 95 percentiles.) Group-wise $\text {E}_{\text {cc,endo}}$ agrees reasonably well across models, while group-wise $\text {E}_{\text {cc,epi}}$ computed with the one-slice Cine model is lower (in magnitude) than group-wise $\text {E}_{\text {cc,epi}}$ computed with the DENSE models

Endocardial and epicardial circumferential strains $\text {E}_{\text {cc}}$ are shown in Fig. 6 for all volunteers and per each mid-ventricular slice separately. $\text {E}_{\text {cc}}$ are reported as median values for the DENSE-based models since multiple strain values are computed per each slice. Circumferential strains computed using the two-slice and one-slice DENSE models match very closely for both endocardial and epicardial values. This is expected since circumferential strains are computed at each slice location and depend on displacements and displacement gradients available on a per slice basis. In addition, the group-wise comparison (Fig. 6, right) shows a reasonably close agreement between the DENSE models and the one-slice Cine model regarding endocardial circumferential strains. (The mean peak systolic $\text {E}_{\text {cc,endo}}$ difference in absolute value is equal to $\approx 1\%$.) The agreement between the DENSE-based models and one-slice Cine model worsens for epicardial circumferential strains: Overall, the one-slice Cine model leads to lower (in magnitude) epicardial strains as reflected in the group-wise comparison plot. (The mean peak systolic $\text {E}_{\text {cc,epi}}$ difference in absolute value is equal to $\approx 7\%.$) The lower magnitude of $\text {E}_{\text {cc,epi}}$ predicted by the one-slice Cine model reflects the limited motion of the LV epicardial surface during contraction and relaxation measured from Cine images.

**Fig. 7:**
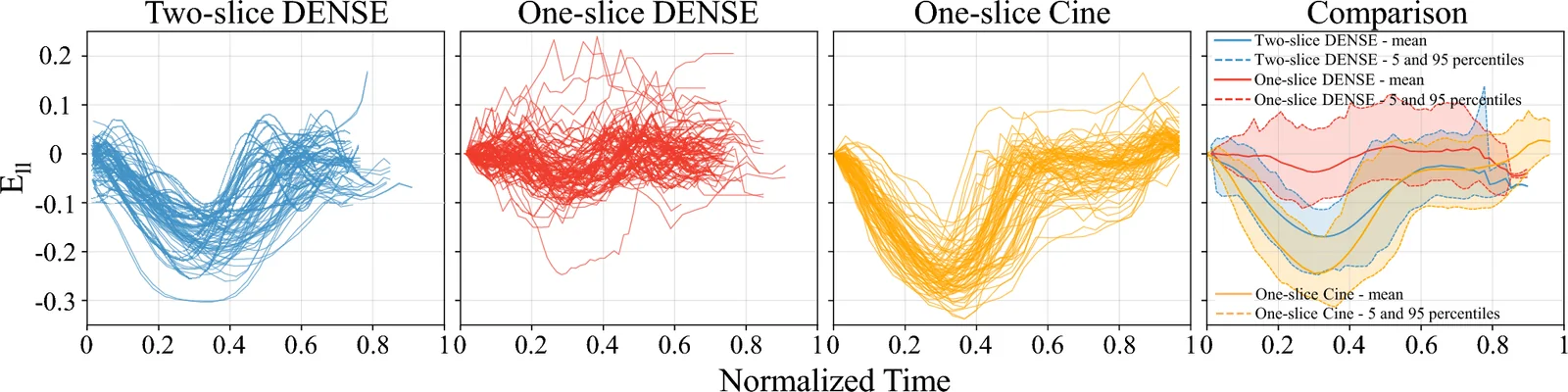
Slice-wise longitudinal strains $\text {E}_{\text {ll}}$ (N=40 subjects) versus normalized time. Each line in the first three columns represents the longitudinal strain per slice obtained (from left to right) with the two-slice DENSE model, one-slice DENSE model, and one-slice Cine model. Median strain values are reported for the DENSE two-slice model as multiple $\text {E}_{\text {ll}}$ values are computed per slice. The mean of all $\text {E}_{\text {ll}}$ curves per model are reported in the right column as solid lines. (Shaded regions represent the range between the 5 and 95 percentiles.) The one-slice Cine model estimates larger (in magnitude) group-wise $\text {E}_{\text {ll}}$ values than the DENSE models and the one-slice DENSE model leads to inaccurate estimates of longitudinal strains

Longitudinal strains $\text {E}_{\text {ll}}$ versus normalized time are illustrated in Fig. 7 per each mid-ventricular slice separately and for all volunteers. Overall, the two-slice DENSE and one-slice Cine models predict clear longitudinal shortening during systole. In contrast, $\text {E}_{\text {ll}}$ computed from the one-slice DENSE model is often non-physiological. This is a consequence of estimating $\text {E}_{\text {ll}}$ from the LVM mask area (Eq. 5): Due to the lower in-plane spatial resolution of DENSE images and the overall lower image contrast with respect to Cine images, DENSE LVM mask segmentations are more sensitive to noise and more prone to error, especially when automated machine-learning (ML) segmentation is adopted as in the current work. Errors in the DENSE LVM mask segmentation propagate directly to the calculation of longitudinal strains, leading to inaccurate estimates. Although the one-slice Cine model is based on the same approach to estimate $\text {E}_{\text {ll}}$, the higher spatial resolution and image contrast lead to more precise LVM masks and, consequently, more physiological $\text {E}_{\text {ll}}$ estimates. Group-wise, peak systolic longitudinal strain computed with the two-slice DENSE model is $\approx -16.9\%$ while the same value estimated from the one-slice Cine model is $\approx -24.4\%$. In contrast, the one-slice DENSE model peak systolic average longitudinal strain is equal to $\approx -4.5\%$, failing to represent the physiological longitudinal shortening at end systole.

**Fig. 8:**
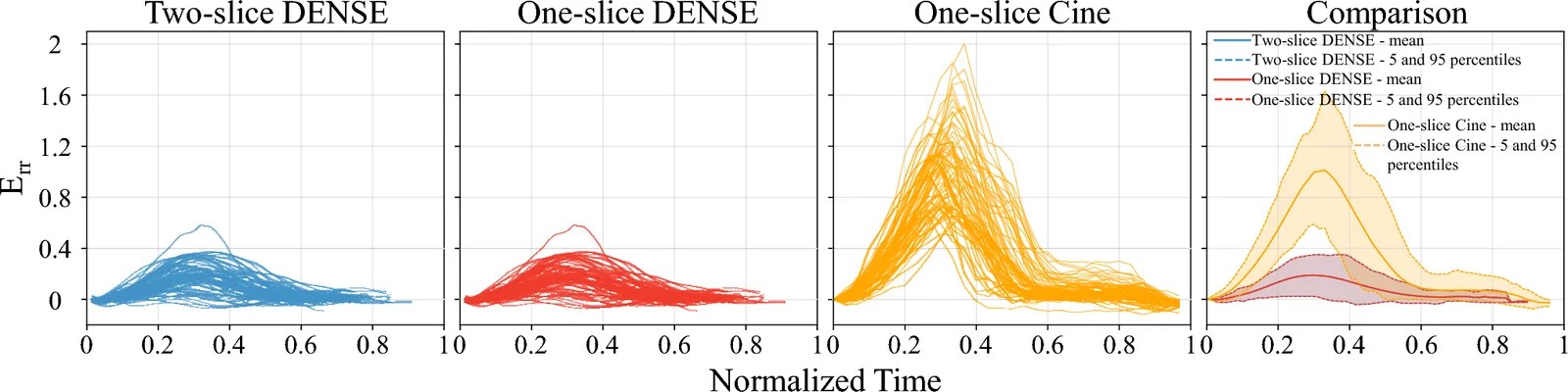
Slice-wise mid-wall radial strains $\text {E}_{\text {rr}}$ (N=40 subjects) versus normalized time. Each line in the first three columns represents the radial strain per slice obtained (from left to right) with the two-slice DENSE model, one-slice DENSE model, and one-slice Cine model. Median strain values are reported for all models as multiple $\text {E}_{\text {rr}}$ values are computed per slice. The mean of all $\text {E}_{\text {rr}}$ curves per model are reported in the right column as solid lines. (Shaded regions represent the range between the 5 and 95 percentiles.) The one-slice Cine model estimates significantly larger group-wise $\text {E}_{\text {rr}}$ values than the DENSE models. This is consistent with the significant wall thickening observed in the Cine images during systole

Figure 8 illustrates slice-wise radial strain $\text {E}_{\text {rr}}$ versus normalized time for all volunteers. Similar to circumferential strains, the two-slice and one-slice DENSE models match closely. Overall, they predict low mid-wall radial strains (average peak systolic group-wise $\text {E}_{\text {rr}}\approx 18.8\%$). Due to the few voxels present across the myocardial wall in DENSE images, the calculation of $\text {E}_{\text {rr}}$ from DENSE data is challenging and sensitive to image noise and processing steps. Automated ML segmentations may also misidentify boundary voxels, further affecting $\text {E}_{\text {rr}}$ calculations. In contrast, slice-wise $\text {E}_{\text {rr}}$ estimated using the one-slice Cine model is significantly larger: Peak systolic mean $\text {E}_{\text {rr}}$ across all volunteers is $\approx 101.3\%$.

**Fig. 9:**
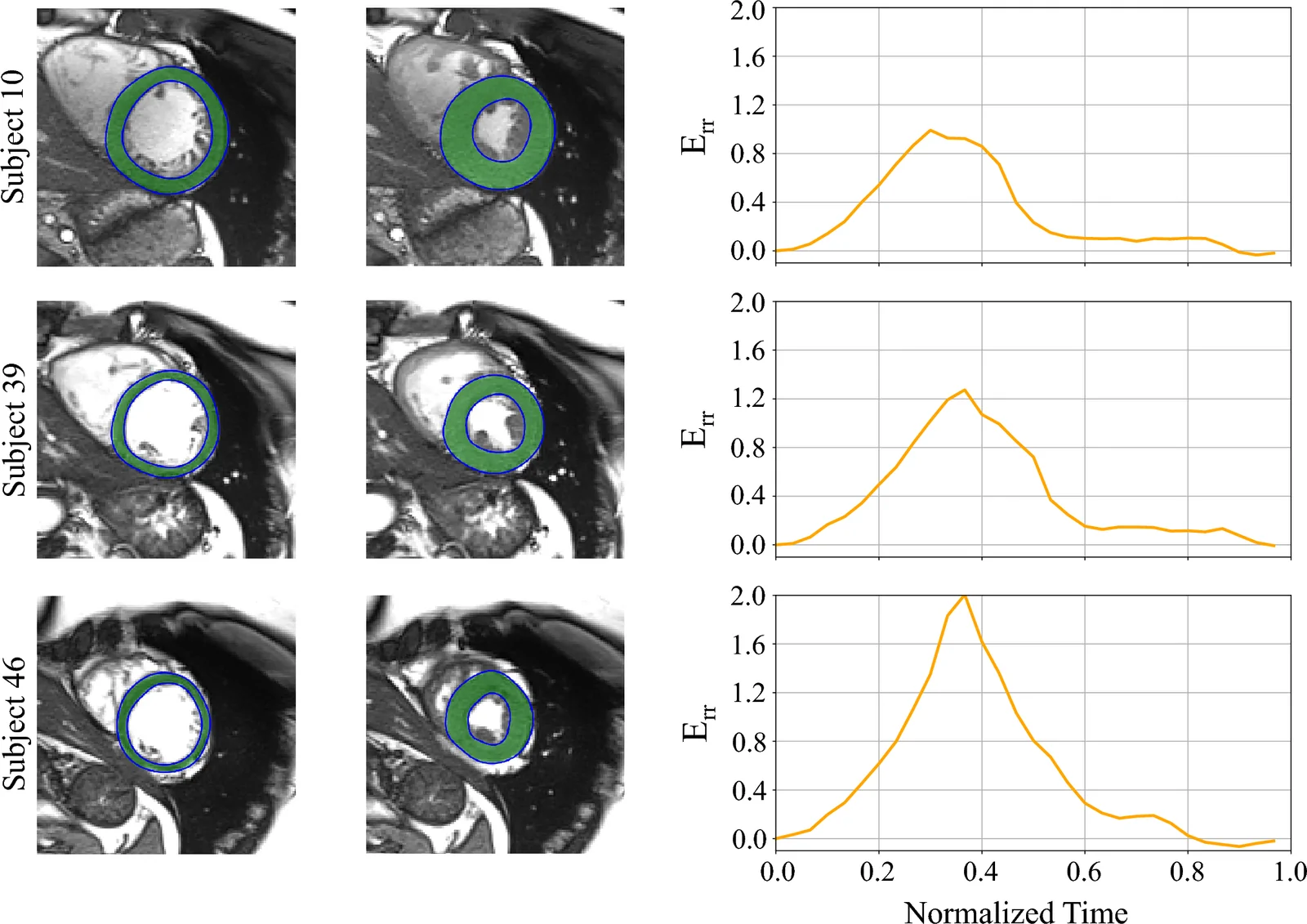
Radial strain computed using the one-slice Cine model. The beginning (reference configuration) and end systole Cine images with their corresponding nnU-Net segmentations and contour splines are shown in the left and middle columns, respectively, for three volunteers (from top to bottom). The epicardial and endocardial contours identified from the LVM masks are used to compute the wall thickness at each cardiac frame and subsequently to estimate $\text {E}_{\text {rr}}$. The large $\text {E}_{\text {rr}}$ estimated from the one-slice Cine model is consistent with the large wall thickening observed in the Cine images from the beginning to the end of systole

The one-slice Cine model estimates $\text {E}_{\text {rr}}$ as a function of wall thickness (in the reference and deformed configurations) and the approximation introduced in Eq. 6. Although this approach is quite sensitive to errors (and/or biases) in image segmentation, it measures directly the wall thickness change during the cardiac cycle. To better understand this approach, we visualize the thickening of the LVM from the beginning of systole (reference configuration) to peak systole in Fig. 9 for three volunteers. The LVM masks superimposed to the original Cine images at the beginning and end of systole show significant thickening, which corresponds to the large radial strain values estimated with the one-slice Cine model. Although consistent with the observed significant wall thickening, $\text {E}_{\text {rr}}$ values computed using the one-slice Cine model may overestimate the true strain values. This may happen, for example, if the trabeculae structure is included in the automatic segmentation at peak systole. A ‘thicker’ segmentation at peak systole would also lead to larger (in magnitude) $\text {E}_{\text {ll}}$ values with respect to the longitudinal strains computed from the two-slice DENSE model based on voxel-wise displacements.

To quantify inter-model strain differences at peak systole across all subjects (N=40 subjects × two slices per subject), paired statistical comparisons are made for each strain component and summarized in Table 1. A significant overall model effect is observed for all strains (p<0.001), indicating systematic differences between the evaluated approaches. Post hoc effect size analysis using Cohen’s *d* demonstrates very good agreement between the two-slice and one-slice DENSE models for both circumferential and radial strain components (d≈0), confirming the consistency in $\text {E}_{\text {cc}}$ and $\text {E}_{\text {rr}}$ obtained using the DENSE-based models (Figs. 6, 7, 8).

The one-slice Cine model exhibits significant differences relative to the two-slice DENSE model for epicardial circumferential strain (d=4.35) and radial strain (d=3.80).

The one-slice Cine model versus the two-slice DENSE model comparison for endocardial circumferential strain yielded a statistically significant difference (p<0.001) with an associated effect size d=-0.89. Although large, this effect size is smaller than those observed for the epicardial circumferential, longitudinal, or radial strain components. Using a selected equivalence margin Δ = 3.5%, the two one-sided tests (TOST) procedure for endocardial circumferential strain obtained with the one-slice Cine and the two-slice DENSE models yields a mean difference of -2.7% (Cine - DENSE) with ${p}_{\text {lower}}<0.05$ and ${p}_{\text {upper}}<0.001$. Since both p-values are less than 0.05, we conclude that the one-slice Cine and two-slice DENSE models are statistically equivalent for endocardial circumferential strains within the chosen margin (Lakens 2017).

A significant difference is also present for $\text {E}_{\text {ll}}$ computed with the one-slice Cine model and the two-slice DENSE model (d=-1.92). This discrepancy is likely due to the methodological difference between the area-based formulation in the one-slice Cine model and the fully 3D DENSE-derived computation of $\text {E}_{\text {ll}}$.

Finally, median [Q1, Q3] pair-wise strain differences at peak systole between the three models are reported in Table 2, confirming: (1) the negligible differences in peak systolic $\text {E}_{\text {cc}}$ and $\text {E}_{\text {rr}}$ computed using the DENSE-based models; (2) the moderate overestimation (in magnitude) of $\text {E}_{\text {cc,endo}}$ computed using the one-slice Cine model versus the DENSE-based models; and (3) the larger discrepancies in the remaining strains across models.

Overall, these findings confirm the qualitative trends shown in Figs. 6, 7, 8.

**Table 1:** Statistical comparison of peak systolic strains (N=40 × 2 slices per subject) across the three models: two-slice DENSE, one-slice DENSE, and one-slice Cine

Strain	Statistical test	Overall *p*	Cine vs. 2-S DENSE *d*	1-S vs. 2-S DENSE *d*
$\text {E}_{\text {cc,epi}}$	RM-ANOVA	< 0.001^†^	4.35	− 0.001
$\text {E}_{\text {cc,endo}}$	RM-ANOVA	< 0.001^†^	− 0.89	0.0002
$\text {E}_{\text {ll}}$	Friedman	< 0.001^‡^	− 1.92	2.42
$\text {E}_{\text {rr}}$	Friedman	< 0.001^‡^	3.80	0.0004

1-S: one-slice; 2-S: two-slice

$^\dagger$Greenhouse–Geisser-corrected repeated-measures ANOVA; $^\ddagger$Friedman Test

Values in comparison columns represent systematic difference quantified by Cohen’s *d* effect size

**Interpretation of **|*d*|: <0.2 (Negligible); 0.2–0.5 (Small); 0.5–0.8 (Medium); >0.8 (Large) (Cohen 2013)

**Table 2:** Per slice pair-wise differences (Median [Q1, Q3]) of peak systolic strains across the proposed models

Strain [%]	Cine vs. 2-S DENSE	1-S vs. 2-S DENSE	Cine vs. 1-S DENSE
$\text {E}_{\text {cc,epi}}$	8.3 [6.6, 10.1]	−0.0 [−0.02, 0.01]	8.3 [6.6, 10.1]
$\text {E}_{\text {cc,endo}}$	−3.0 [−5.2, 0.1]	−0.0 [−0.01, 0.01]	−3.0 [−5.2, 0.1]
$\text {E}_{\text {ll}}$	−8.6 [−12.4, −5.2]	12.9 [8.6, 16.7]	−21.6 [−24.6, −17.0]
$\text {E}_{\text {rr}}$	83.6 [66.2, 104.9]	0.0 [−0.01, 0.02]	83.6 [66.2, 104.9]

Strains are reported in %

1-S: one-slice; 2-S: two-slice

**Fig. 10:**
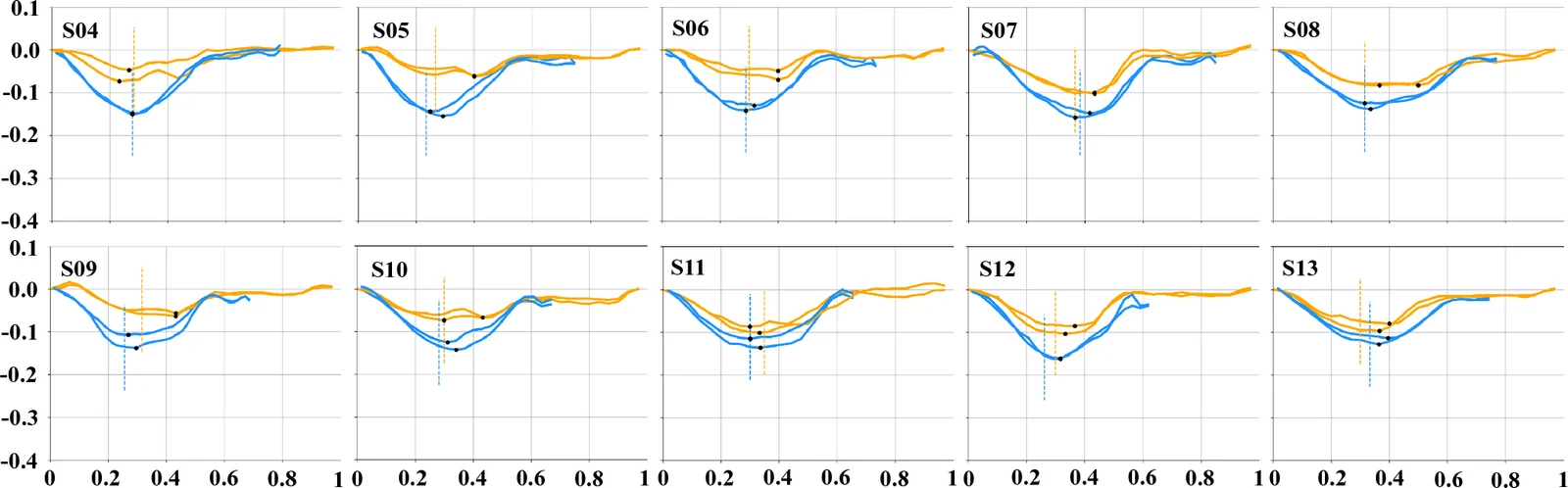
Epicardial circumferential strains ($\text {E}_{\text {cc,epi}}$) versus normalized time computed for ten subjects using the two-slice DENSE model (blue lines) and the one-slice Cine model (orange lines). For each model, each line corresponds to an individual slice. The average frame per model with the smallest left ventricular cavity (LVC) is marked with a dashed vertical line while the minimum $\text {E}_{\text {cc,epi}}$ values per slice are marked with black circular markers. Epicardial circumferential strains computed from the two-slice DENSE model are in general larger (in magnitude) than the corresponding strain values computed from the one-slice Cine model

**Fig. 11:**
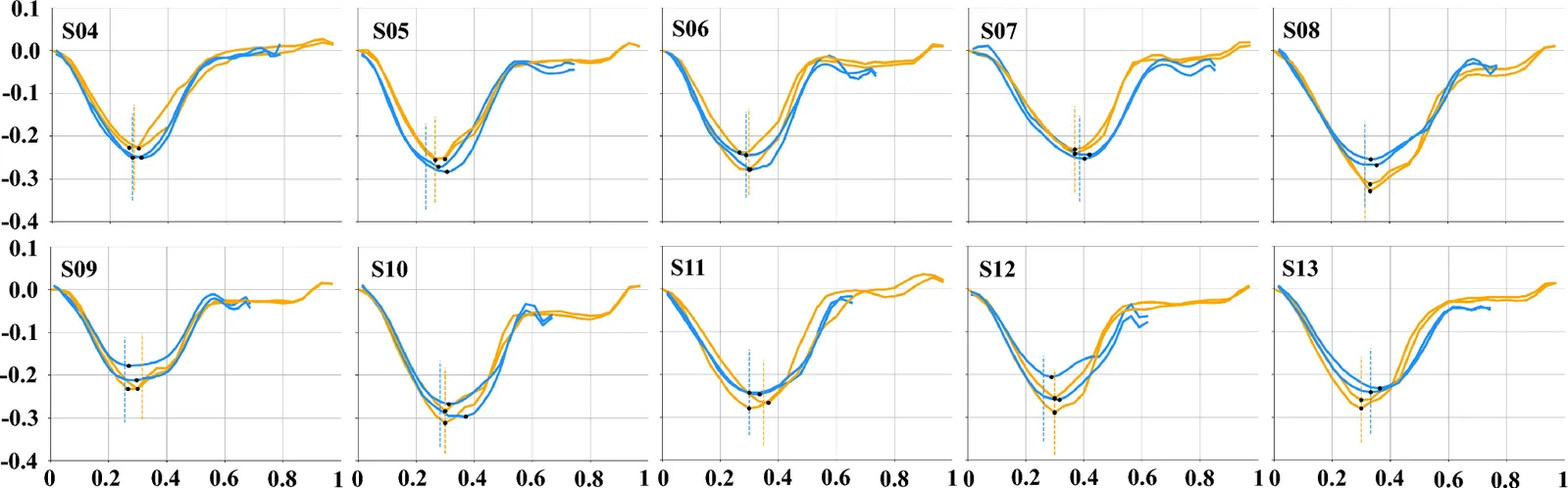
Endocardial circumferential strains ($\text {E}_{\text {cc,endo}}$) versus normalized time computed for ten subjects using the two-slice DENSE model (blue lines) and the one-slice Cine model (orange lines). For each model, each line corresponds to an individual slice. The average frame per model with the smallest left ventricular cavity (LVC) is marked with a dashed vertical line while the minimum $\text {E}_{\text {cc,endo}}$ values per slice are marked with black circular markers. Endocardial circumferential strains computed from the two-slice DENSE model and one-slice Cine model are in overall good agreement both regarding their temporal evolution and in magnitude largest (peak systolic) value

**Fig. 12:**
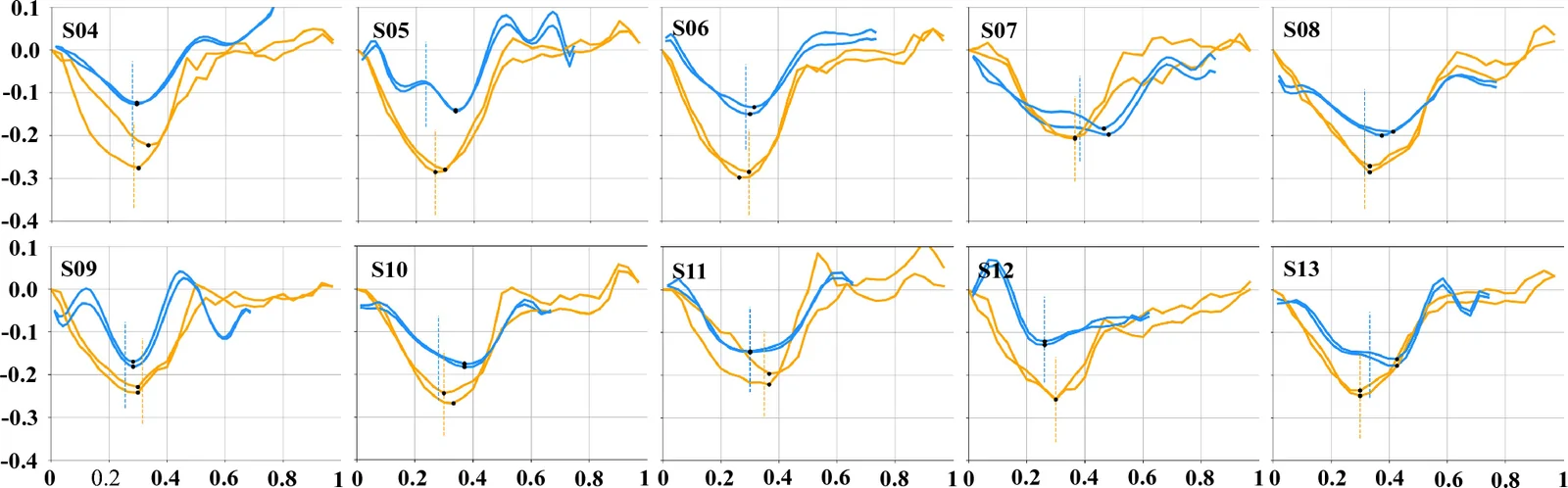
Longitudinal strains ($\text {E}_{\text {ll}}$) versus normalized time computed for ten subjects using the two-slice DENSE model (blue lines) and the one-slice Cine model (orange lines). For each model, each line corresponds to an individual slice. The average frame per model with the smallest left ventricular cavity (LVC) is marked with a dashed vertical line while the minimum $\text {E}_{\text {ll}}$ values per slice are marked with black circular markers. With respect to the two-slice DENSE model, longitudinal strains computed from the one-slice Cine model show smaller oscillations with respect to normalized time and higher (in magnitude) peak systolic values

**Fig. 13:**
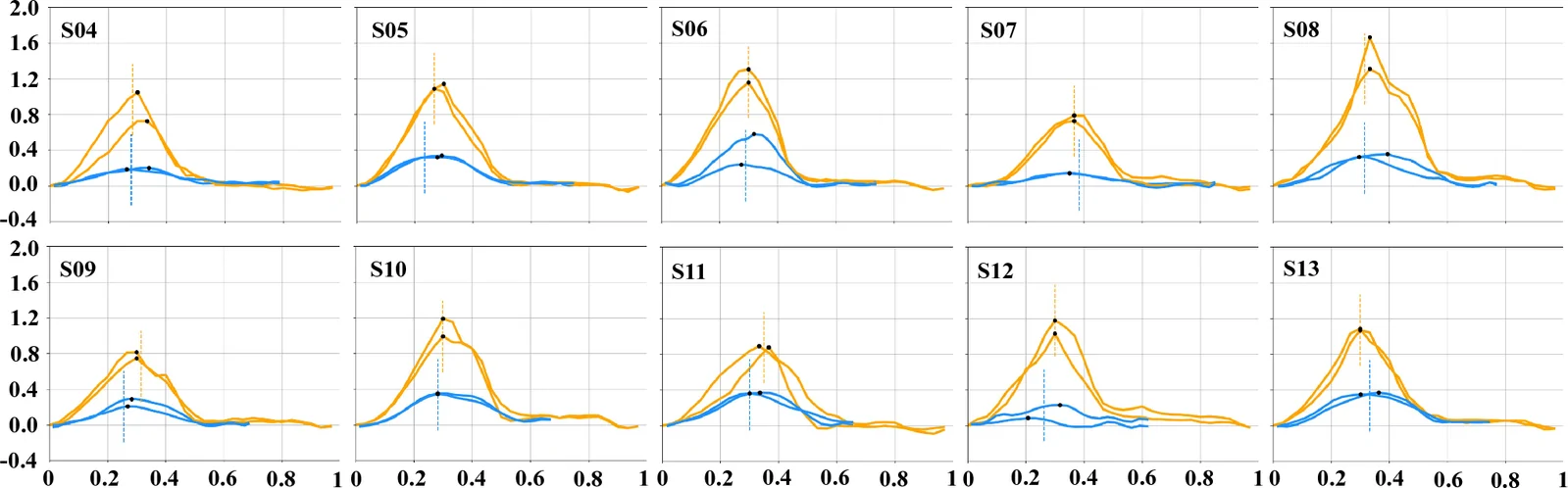
Radial strains ($\text {E}_{\text {rr}}$) estimated at mid-wall versus normalized time computed for ten subjects using the two-slice DENSE model (blue lines) and the one-slice Cine model (orange lines). For each model, each line corresponds to an individual slice. The average frame per model with the smallest left ventricular cavity (LVC) is marked with a dashed vertical line while the maximum $\text {E}_{\text {rr}}$ values per slice are marked with black circular markers. Radial strains predicted from the one-slice Cine model are significantly larger than values predicted from the two-slice DENSE model

To evaluate the proposed models on an individual subject basis, we compare the strains computed from the two-slice DENSE model with the ones estimated from the one-slice Cine model. Both mid-ventricular slices are plotted separately and median values are reported for the strains computed using the two-slice DENSE model. Findings are consistent across all volunteers, and strains plots for the first ten subjects are shown in Figs. 10, 11, 12, 13, while the strains for the remaining volunteers are shown in Appendix A (Figs. 15, 16, 17, 18).

In general, individual volunteer strain plots are in agreement with group-wise findings. Endocardial circumferential strains computed using the two-slice DENSE model and one-slice Cine model agree well throughout the cardiac cycle and endocardial $\text {E}_{\text {cc}}$ remains the strain component that is more robustly estimated across all models. In terms of the other strain components, larger (in magnitude) epicardial circumferential strains, and smaller (in magnitude) longitudinal and radial strains are predicted by the two-slice DENSE model throughout the cardiac cycle when compared to the values estimated by the one-slice Cine model.

In addition to the strain magnitudes discussed above, we notice significant differences also in the strain temporal variation predicted by the two-slice DENSE and one-slice Cine models. The overall high quality of the strain traces obtained with the one-slice Cine model allows to detect the initial increase in strain magnitude during systole, followed by a decrease corresponding to rapid filling and a plateau phase during diastole, before a final strain change during atrial systole. These phases are more clearly visible in circumferential and radial strains than in longitudinal strain plots. The latter are more susceptible to slight changes in LVM segmented area and therefore $\text {E}_{\text {ll}}$ plots presents more oscillations. In contrast, strain plots computed from the DENSE-based models do not show the same level of temporal details: (1) DENSE acquisition often does not cover the full cardiac cycle, preventing the evaluation of strains during the entire duration of diastasis and atrial systole; (2) due to the chosen DENSE data processing strategy, oscillations are present, especially in the frames toward the end of the DENSE acquisition window; and (3) significant $\text {E}_{\text {ll}}$ oscillations are occasionally observed (e.g., in subjects S05 and S09), likely due to low SNR, e.g., in early systole or late diastole.

### One-slice Cine model strain sensitivity to image segmentation

The strains estimated using the one-slice Cine model depend on the segmentation mask. To evaluate the sensitivity of the computed strains on variations/uncertainty of the Cine slice segmentation, two tests are carried out: (1) The original segmentation computed using the nnU-Net model trained on the current dataset is modified by changing the default threshold ($p_{\text {LVM}}\ge 0.5$) employed to generate the LVM mask; and (2) the segmentations of the Cine slices in the current dataset are obtained using a different nnU-Net model trained on the publicly available ACDC dataset (Bernard et al. 2018).

#### Variation in LVM masks due to different probability thresholds

The nnU-Net model can provide directly the LVM binary mask or a probability mask holding per-pixel LVM probabilities. The binary LVM mask can be computed from the probability mask by selecting all the pixels with LVM probability $p_{\text {LVM}}\ge 0.5$. Using the probability mask instead of the default binary mask, a different threshold to identify a pixel as LVM can be selected: Lowering the threshold probability will add pixels to the LVM mask (leading to a larger, ‘more permissive’ mask), while increasing the probability threshold will remove pixels from the LVM mask (leading to a smaller, ‘more conservative’ mask). In this test, we created variation of the original binary masks (obtained with $p_{\text {LVM}}\ge 0.5$) using a lower and a higher threshold equal to 0.05 and 0.95, respectively (i.e., the LVM mask is created selecting all the pixels with $p_{\text {LVM}}\ge 0.05$ or $p_{\text {LVM}}\ge 0.95$, respectively). According to this approach, pixels are added or removed based on the ‘uncertainty of the model’ which changes at each location and time frame, i.e., pixels are not added or removed in the same location at each frame. The average DICE similarity coefficient corresponding to all perturbed LVM masks with respect to the original LVM binary masks is ≈0.97. The peak systolic strains and peak systolic strain differences (reported as absolute values) computed using the original and perturbed masks are reported in Table 3. The results show small strain differences for $\text {E}_{\text {cc,epi}}$ and $\text {E}_{\text {cc,endo}}$ (median absolute differences $\lessapprox 0.5\%$) and $\text {E}_{\text {ll}}$ (median absolute differences $\lessapprox 1\%$). Peak systolic median absolute $\text {E}_{\text {rr}}$ differences across segmentations are larger (≈4-4.5%) highlighting the higher sensitivity of $\text {E}_{\text {rr}}$ to small variations in LVM wall thickness. Overall, adding pixels ($p_{\text {LVM}}\ge 0.05$) lowered $\text {E}_{\text {rr}}$ while removing pixels ($p_{\text {LVM}}\ge 0.95$) increased $\text {E}_{\text {rr}}$ with respect to $\text {E}_{\text {rr}}$ computed using the default mask ($p_{\text {LVM}}\ge 0.5$). This result is due to changes in LVM thickness in both the reference and peak systolic configurations.

**Fig. 14:**
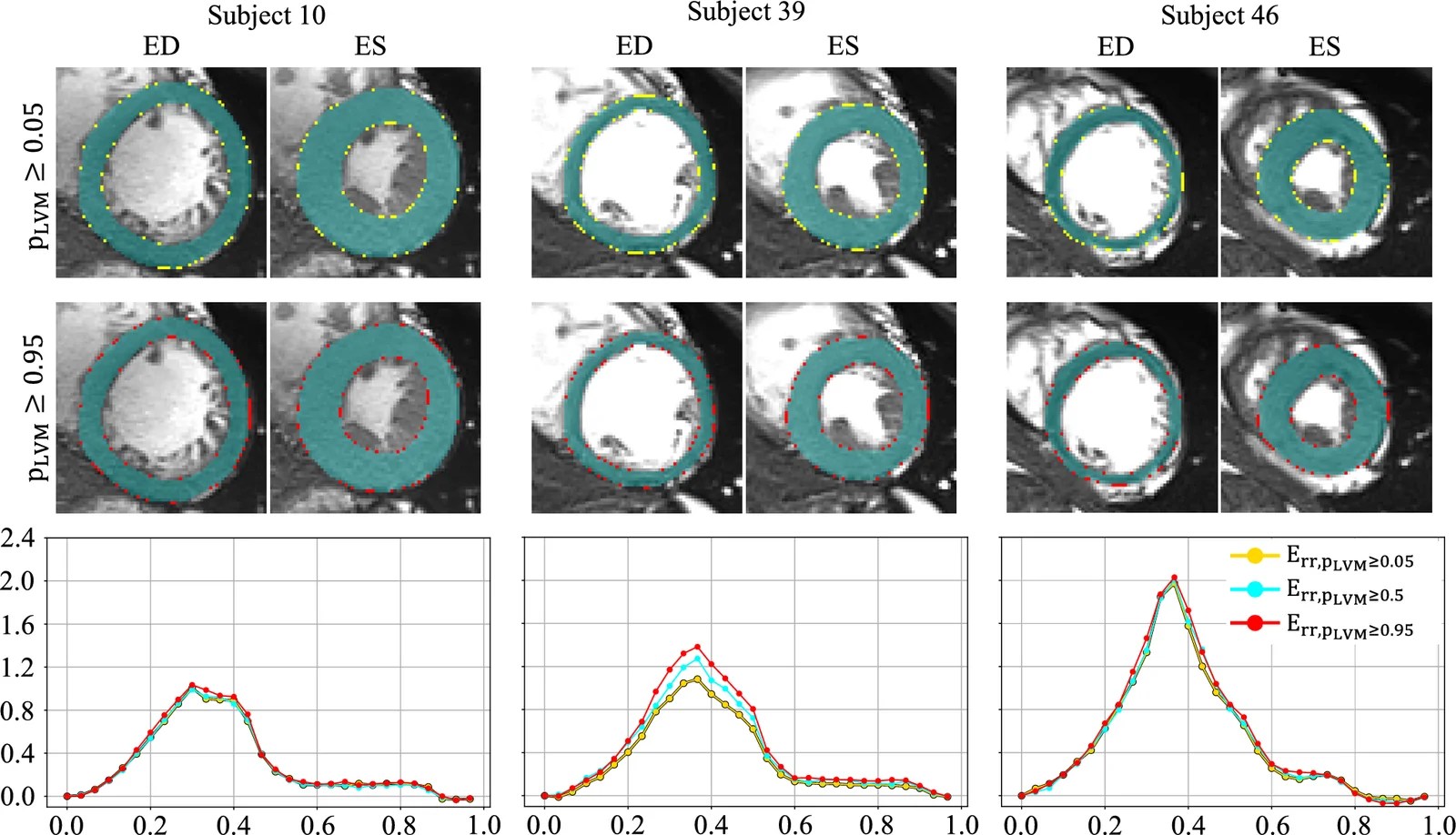
Perturbation of the LVM masks and corresponding radial strains throughout the cardiac cycle obtained using the one-slice Cine model. Variations of the LVM masks are created by selecting all pixels with $p_{\text {LVM}}\ge 0.05$ or $p_{\text {LVM}}\ge 0.95$. The former results in adding pixels while the latter results in removing pixels from the original segmentation obtained using $p_{\text {LVM}}\ge 0.5$. Top and middle rows: added (marked in yellow) and removed (marked in red) pixels are shown in representative end-diastolic (ED) and end-systolic (ES) LVM masks. Bottom row: median radial strains throughout the cardiac cycle corresponding to the original and perturbed masks

**Fig. 15:**
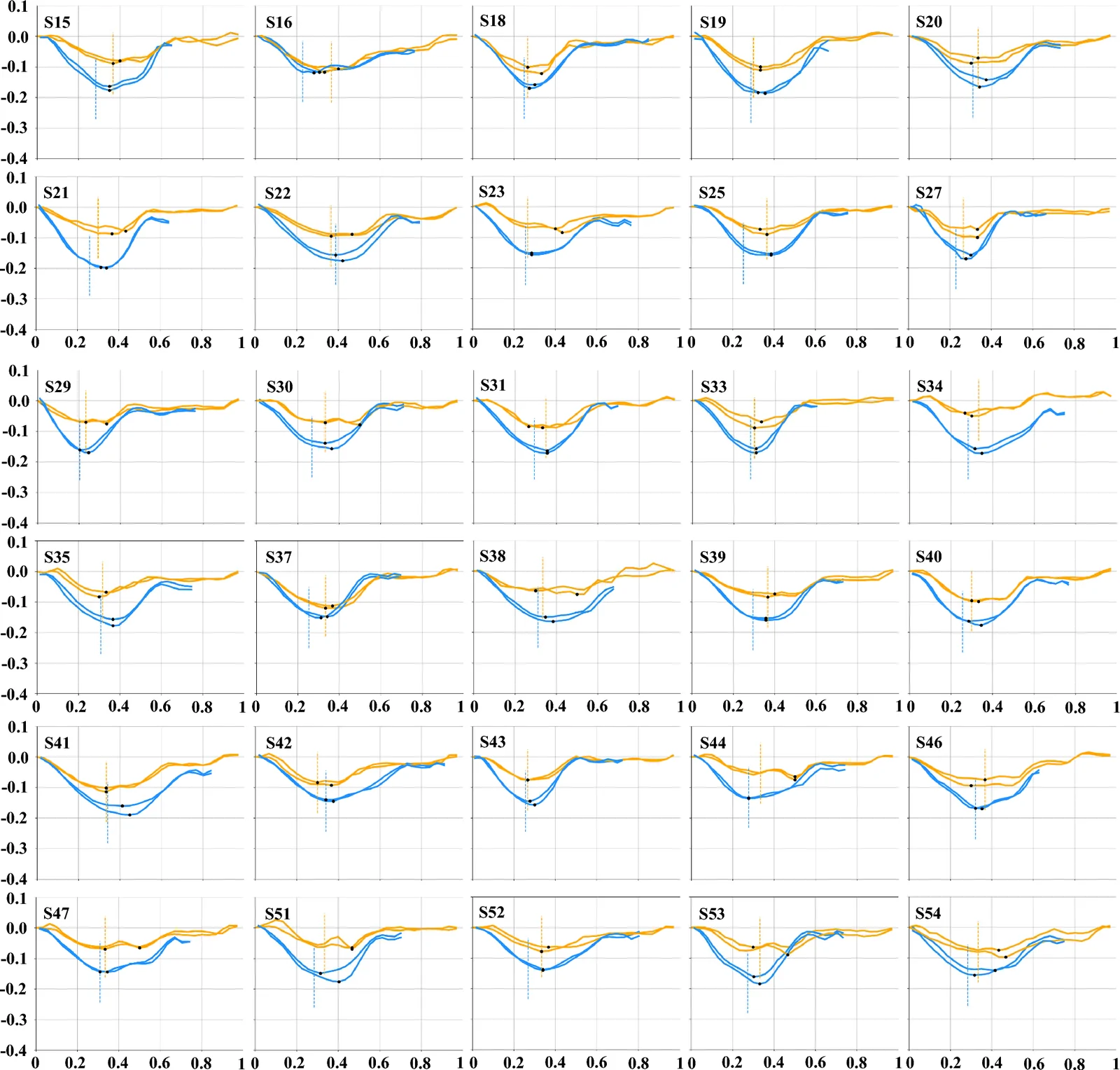
Epicardial circumferential strains ($\text {E}_{\text {cc,epi}}$) versus normalized time computed for the remaining thirty subjects not shown in the main text using the two-slice DENSE model (blue lines) and the one-slice Cine model (orange lines). For each model, each line corresponds to an individual slice. The average frame per model with the smallest left ventricular cavity (LVC) is marked with a dashed vertical line while the minimum $\text {E}_{\text {cc,epi}}$ values per each slice are marked with black circular markers. Overall, the one-slice Cine model predicts lower (in magnitude) epicardial slice-wise circumferential strains than the two-slice DENSE model

**Fig. 16:**
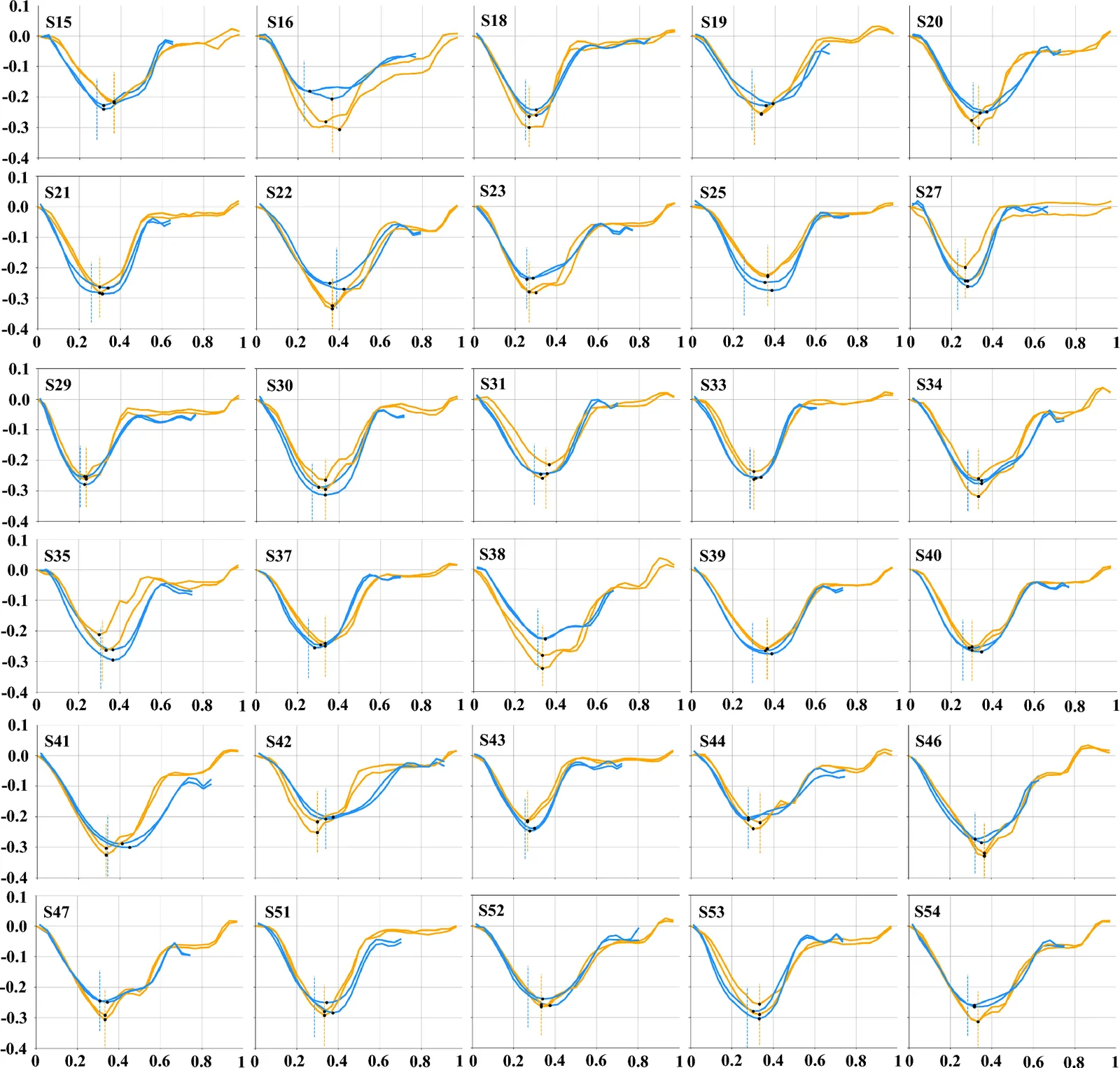
Endocardial circumferential strains ($\text {E}_{\text {cc,endo}}$) versus normalized time computed for the remaining thirty subjects not shown in the main text using the two-slice DENSE model (blue lines) and the one-slice Cine model (orange lines). For each model, each line corresponds to an individual slice. The average frame per model with the smallest left ventricular cavity (LVC) is marked with a dashed vertical line while the minimum $\text {E}_{\text {cc,endo}}$ values per slice are marked with black circular markers. The two-slice DENSE model and one-slice Cine model continue to agree reasonably well in all subjects

**Fig. 17:**
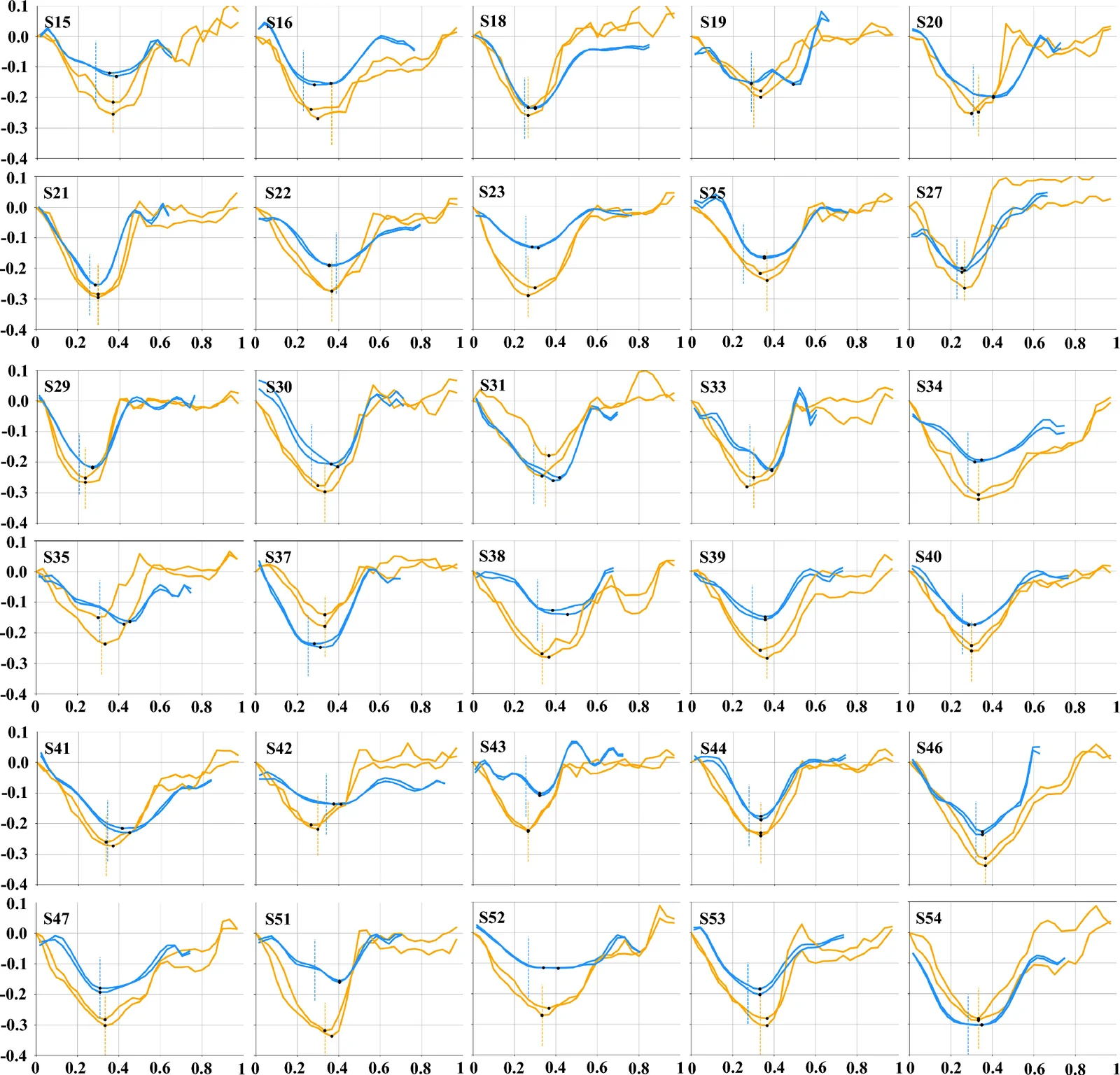
Longitudinal strains ($\text {E}_{\text {ll}}$) versus normalized time computed for the remaining thirty subjects not shown in the main text using the two-slice DENSE model (blue lines) and the one-slice Cine model (orange lines). For each model, each line corresponds to an individual slice. The average frame per model with the smallest left ventricular cavity (LVC) is marked with a dashed vertical line while the minimum $\text {E}_{\text {ll}}$ values per slice are marked with black circular markers. Overall, the one-slice Cine model predicts larger (in magnitude) slice-wise longitudinal strains than the two-slice DENSE model

**Fig. 18:**
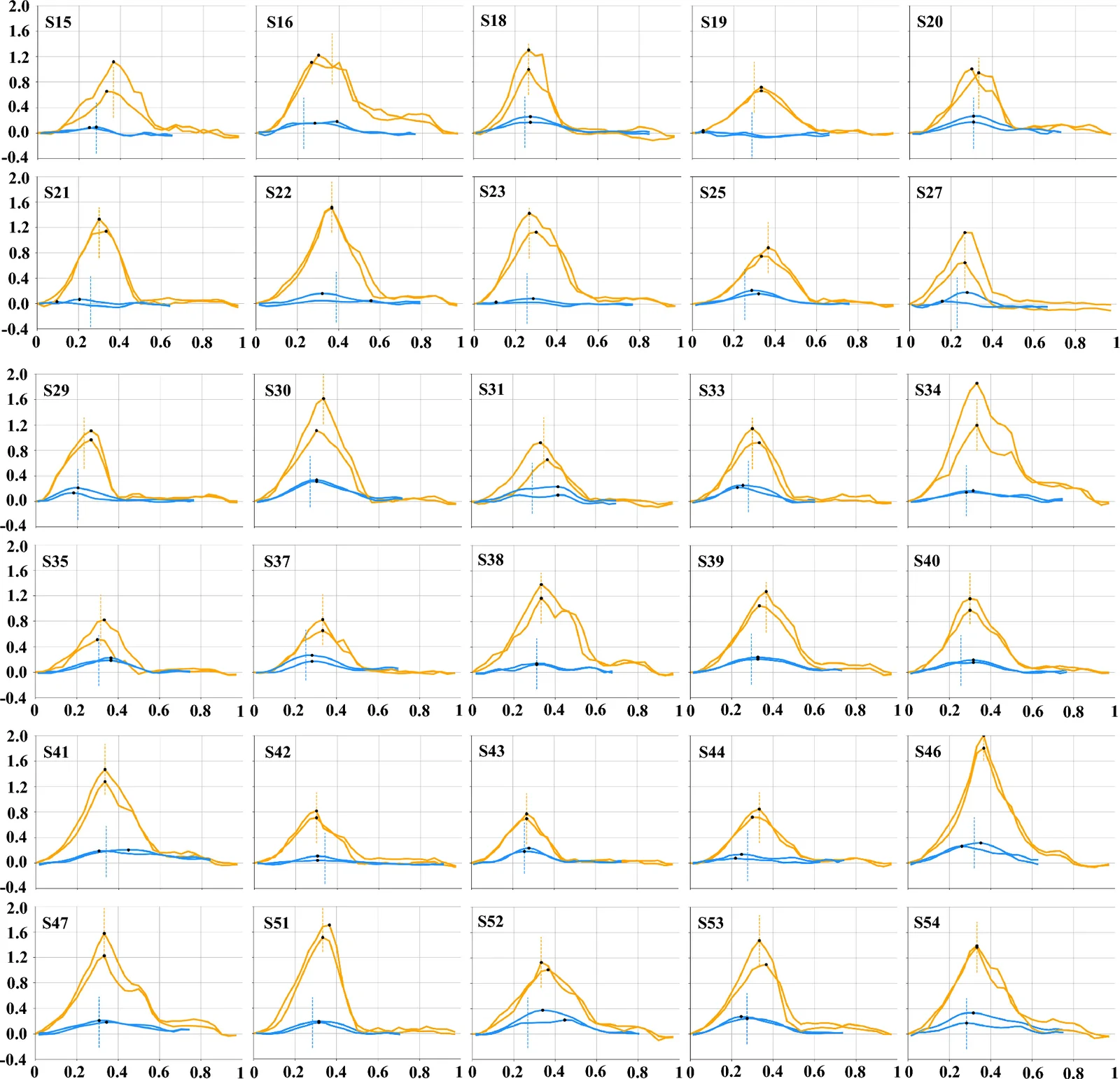
Radial strains ($\text {E}_{\text {rr}}$) estimated at mid-wall versus normalized time computed for the remaining thirty subjects not shown in the main text using the two-slice DENSE model (blue lines) and the one-slice Cine model (orange lines). For each model, each line corresponds to an individual slice. The average frame per model with the smallest left ventricular cavity (LVC) is marked with a dashed vertical line while the largest $\text {E}_{\text {rr}}$ values per slice are marked with black circular markers. The one-slice Cine model predicts significantly larger slice-wise radial strains than the two-slice DENSE model

Representative examples of the changes in LVM masks obtained with the described approach are shown in Fig. 14, together with the corresponding $\text {E}_{\text {rr}}$ curves throughout the cardiac cycle.

These results suggest that strains computed using the one-slice Cine model are robust with respect to small perturbations of the LVM masks.

**Table 3:** Peak systolic strain values (median [Q1, Q3]) obtained using the original ($p_{\text {LVM}}\ge 0.5$) and perturbed ($p_{\text {LVM}}\ge 0.05$ or $p_{\text {LVM}}\ge 0.95$) LVM masks. Strain differences (median [Q1, Q3]) are computed in absolute value

Strain [%]	${p_{\text {LVM}}\ge 0.5}$	${p_{\text {LVM}}\ge 0.05}$	${p_{\text {LVM}}\ge 0.95}$	$|\Delta _{0.5-0.05}|$	$|\Delta _{0.5-0.95}|$
$\text {E}_{\text {cc,epi}}$	−7.5 [−9.3, −6.4]	−7.5 [−9.2, −6.3]	−7.8 [−9.2, −6.6]	0.1 [0.0, 0.2]	0.1 [0.1, 0.2]
$\text {E}_{\text {cc,endo}}$	−26.3 [−29.2, −24.1]	−26.4 [−29.5, −24.3]	−26.0 [−28.8, −24.1]	0.2 [0.1, 0.3]	0.3 [0.1, 0.4]
$\text {E}_{\text {ll}}$	−25.8 [−28.1, −22.9]	−25.2 [−27.4, −22.1]	−26.6 [−28.9, −23.5]	0.7 [0.5, 1.2]	0.8 [0.3, 1.2]
$\text {E}_{\text {rr}}$	107.2 [82.1, 129.6]	101.6 [81.0, 119.0]	112.7 [85.3, 136.0]	3.9 [2.1, 7.7]	4.4 [1.8, 8.4]

Strains are reported in %

#### Variation in LVM masks due to different segmentation models

A new model was trained using the ACDC dataset (Bernard et al. 2018) to evaluate the one-slice Cine model strain sensitivity to segmentation models. The ACDC dataset consists of 150 subjects, of which 100 are used for training and validation and 50 for testing. The new model was trained using a fivefold cross-validation approach using images from 80 subjects for training and images from 20 subjects for validation following the strategy discussed in Isensee et al. (2017). The model trained on the ACDC dataset achieved an LVM DICE coefficient of ≈0.87 when tested on the healthy volunteers dataset acquired for this study (consisting of ≈1200 images and including apical and basal slices) and a LVM DICE coefficient of ≈0.90 when evaluated on the ACDC’s testing set comprised of the 50 subjects not used during training.

All strains were recomputed using the LVM masks obtained with the segmentation model trained using the ACDC dataset and the absolute per slice strain differences at peak systole are reported in Table 4 as mean [SD] and median [Q1, Q3]. We notice small median/mean differences in circumferential ($\lessapprox 1.5\%$) and longitudinal ($\lessapprox 2\%$) strains and a larger median/mean difference in radial strains ($\lessapprox 15\%$), which however corresponds to a larger overall strain value.

These results suggest that the strains, especially circumferential and longitudinal strains, computed using the one-slice Cine model are robust with respect to variations in LVM segmentation models, although a broader study including more segmentation models is needed.

**Table 4:** One-slice Cine model peak systolic strains computed using the nnU-Net segmentation model trained on this study data (labeled ‘This Study’) and the nnU-Net segmentation model trained on the ACDC dataset (labeled ‘ACDC’)

Strain [%]	This study	ACDC	$|{\rm{This\; study - ACDC}}|$
**Mean [SD]**			
$\text {E}_{\text {cc, epi}}$	-7.8[1.9]	-8.9[1.9]	1.2[0.9]
$\text {E}_{\text {cc, endo}}$	-26.7[3.3]	-27.4[3.3]	0.9[0.7]
$\text {E}_{\text {ll}}$	-25.4[3.9]	-25.1[3.6]	2.0[1.9]
$\text {E}_{\text {rr}}$	109.2[31.5]	110.8[31.9]	14.2[11.5]
**Median [Q1, Q3]**			
$\text {E}_{\text {cc, epi}}$	-7.5[-9.3,-6.4]	-8.8[-10.4,-7.8]	1.0[0.6,1.6]
$\text {E}_{\text {cc, endo}}$	-26.3[-29.2,-24.1]	-27.0[-30.0,-24.6]	0.7[0.4,1.3]
$\text {E}_{\text {ll}}$	-25.8[-28.1,-22.9]	-25.4[-27.8,-22.6]	1.5[0.6,2.9]
$\text {E}_{\text {rr}}$	107.2[82.1,129.6]	104.3[84.8,135.2]	10.7[5.0,19.6]

Strains and strain differences are reported in [%] as mean [SD] and median [Q1, Q3]. Strain difference metrics are computed by first evaluating the absolute strain differences per slice

### Current limitations and future work

Several aspects in the presented models can be improved in future developments. First, both DENSE and Cine models are based on the automated segmentation of the LVM using an nnU-Net model. This feature results in significantly less processing time to compute cardiac strains. Increasing the size and diversity of the training dataset will strengthen the robustness of the LVM segmentations and consequently improve the strain estimates. Ways of diversifying the training data include adding images acquired in patients with various cardiomyopathies as well as data acquired with different sequence parameters and scanner hardware. This is particularly relevant for the DENSE image segmentation, which, due to lower image contrast, is often more challenging than Cine image segmentation. Furthermore, voxels misclassified during segmentation, especially at the endocardial boundary, can lead to significant error propagation in the subsequent displacement and strain calculations. Without additional training and testing including images acquired in patients affected by cardiomyopathies, the proposed segmentation models and strain calculation pipelines may not generalize well to data with abnormal heart anatomy and/or wall motion.

Second, and related to the previous aspect, differently trained ML models may consistently segment Cine images with a bias introduced by the segmentations in the training dataset (e.g., different observers may segment the LVM region more or less conservatively depending on their training and selected principles to guide the segmentations). These biases propagate to the computed strains and may complicate strain comparison across studies in which different ML architectures and training datasets are used. As discussed previously, using large datasets including segmentations from multiple observers mitigates this risk.

Third, due to its wide use and public availability, we adopted the DENSE-Analysis toolbox to compute voxel-wise displacements from the DENSE segmented images. However, newer methods have been proposed to improve displacement (and subsequently strain) calculations from DENSE imaging data (e.g., Ghadimi et al. 2021, 2023). Adopting these newer schemes may improve the results obtained from the two-slice and one-slice DENSE models, especially regarding longitudinal and radial strains, which are more sensitive to noise.

Fourth, $\text {E}_{\text {cc,epi}}$, $\text {E}_{\text {cc,endo}}$, and $\text {E}_{\text {ll}}$ computed using the one-slice Cine model, and $\text {E}_{\text {ll}}$ computed using the one-slice DENSE model are ‘per slice’ values and cannot be evaluated regionally, e.g., per AHA region (Cerqueira et al. 2002) or, regarding $\text {E}_{\text {ll}}$, also transmurally. This aspect prevents their use to study regional abnormalities and regional motion patterns in the short-axis slice plane.

Fifth, the longitudinal strain calculation in the one-slice DENSE and Cine models is based on the approximation that the myocardium volume per slice is preserved. This approximation can be avoided by including long-axis slices, from which longitudinal strains could be directly computed. In the case of Cine MRI acquisition, long-axis slices are routinely acquired in clinical exams facilitating their future inclusion.

Finally, we note that, while our phantom model allows to compare the strains computed using the proposed models to ground-truth analytical values, it is based on a cylindrical geometry and implements a simplified motion. A phantom (e.g., Mukherjee et al. 2024) with a more complex anatomy and motion (regionally and transmurally) will allow to complement the validation of the proposed models. Furthermore, the phantom adopted in this study was specifically developed to generate virtual DENSE images, whose magnitude components were used here also as an approximation of Cine images. A phantom model specifically developed to generate Cine MR images will lead to a more robust and complete evaluation of the one-slice Cine model, especially at different SNR levels.

## Conclusions

Building upon the framework and results initially presented at the FIMH 2025 conference (Marques et al. 2025), in this study we discussed three models. These models are based on automated LVM segmentations to compute cardiac strains using progressively less motion information, from the full voxel-wise displacement field of the two-slice DENSE model to only the motion of the LVM contours in the one-slice Cine model. All three models agree reasonably well in terms of computed endocardial circumferential strains, but significant differences persist in longitudinal and radial strains. Based on both the computational phantom and volunteer results, the DENSE-based models may significantly underestimate overall radial strains, especially toward the endocardium. Furthermore, the longitudinal strains approximated using the one-slice DENSE model are affected by substantial errors due to larger uncertainty in the DENSE image segmentation. With respect to the two-slice DENSE model, the one-slice Cine model leads to lower (in magnitude) epicardial circumferential strains and significantly larger radial strains. The lower epicardial circumferential strains are consistent with limited epicardial wall motion, while the significantly larger radial strains are consistent with the substantial wall thickening measured from the automatic segmentation of the myocardial wall. The strain plots versus normalized time computed from the one-slice Cine model show expected physiological features, such as a plateau corresponding to diastasis and a change in strain magnitude corresponding to atrial systole. These features support the viability of the one-slice Cine model. Although further improvements and testing are necessary, the one-slice Cine model has the potential to provide simple estimates of slice-wise circumferential, longitudinal, and radial strains from contour motion only and can be readily implemented in a clinical workflow where Cine MR images are routinely acquired. The automated LVM segmentations and strain calculations further facilitate its implementation to process large datasets quickly or individual subjects’ images in real time. Additionally, the one-slice Cine model can be applied to short-axis images acquired using other modalities, such as echocardiography and computed tomography, from which single slice contour motion can be extracted.

## Data Availability

Cine and DENSE MRI data are available at https://purl.stanford.edu/xt223hz2438. The code produced in this research is publicly available on GitHub at the links provided in the method section.

## Appendix A: Individual strain plots obtained with the two-slice DENSE and one-slice Cine models

In the following, we report the slice-wise circumferential (epicardial and endocardial), longitudinal, and radial strains versus normalized time computed using the two-slice DENSE and one-slice Cine models for the remaining subjects not shown in the main text (Figs. 10, 11, 12, 13). The subjects are divided based on numerical order, with the strains computed for the first ten subjects shown in the main text and the strains computed for the remaining thirty subjects shown in this appendix. Although strain values and models’ agreement vary per subject, the same main features highlighted in Figs. 10, 11, 12, 13 are visible in Figs. 15, 16, 17, 18.

## Declarations

### Code availability

The main code developed for this research is freely available at https://github.com/CBL-UCF/SingleSliceCardiacStrains.
